# Genomic analysis and chitinase characterization of *Vibrio harveyi* WXL538: insight into its adaptation to the marine environment

**DOI:** 10.3389/fmicb.2023.1121720

**Published:** 2023-07-03

**Authors:** Lingman Ran, Xiaolei Wang, Xinxin He, Ruihong Guo, Yanhong Wu, Pingping Zhang, Xiao-Hua Zhang

**Affiliations:** ^1^Frontiers Science Center for Deep Ocean Multispheres and Earth System, College of Marine Life Sciences, Ocean University of China, Qingdao, China; ^2^Laboratory for Marine Ecology and Environmental Science, Laoshan Laboratory, Qingdao, China; ^3^Institute of Evolution & Marine Biodiversity, Ocean University of China, Qingdao, China

**Keywords:** genome analysis, *Vibrio harveyi*, chitinase characterization, adaptation, marine environment

## Abstract

Chitin, the most abundant bio-polymer in seawater, may be utilized by various microorganisms as a carbon source. Vibrios have been regarded as one of the main groups of chitin consumers in the marine carbon cycle and chitinase producers. The organisms are widely distributed in the aquatic environment. However, the co-working mechanism between their chitinases, and whether the chitinase’s diversity contributes to their adaption to the environment, needs to be further elucidated. Here, we obtained a chitinolytic strain, *Vibrio harveyi* WXL538 with eight putative chitinase-coding genes. Five of the genes, i.e., Chi4733, Chi540, Chi4668, Chi5174, and Chi4963, were overexpressed and validated, in which Chi4668, Chi4733 and Chi540 were purified and characterized. The result of Chi4668 was described in our previous study. Endo-chitinase Chi4733 degraded colloidal chitin to produce (GlcNAc)_2_ and minor (GlcNAc)_3_. The enzymatic activity of Chi4733 was 175.5 U mg^−1^ and *K*cat/*K*m was 54.9 s^−1^ M^−1^. Chi4733 had its maximum activity at 50°C and pH 4–6, activated by Sr^2+^, Co^2+^, Ca^2+^, and Mg^2+^ and inhibited by Al^3+^, Zn^2+^, Cu^2+^, Ni^2+^, and SDS. Exo-chitinase Chi540 degraded colloidal chitin to (GlcNAc)_2_. The enzymatic activity of Chi540 was 134.5 U mg^−1^ and *K*cat/*K*m was 54.9 s^−1^ M^−1^. Chi540 had its maximum activity at 60°C and pH 6–8, was activated by Sr^2+^, Ca^2+^, and Mg^2+^ but inhibited by K^+^, Ba^2+^, Zn^2+^, Cu^2+^, Ni^2+^, SDS and urea. Whole genome analysis of *V. harveyi* WXL538 and characterization of its chitinase can provide a better understanding of its adaptability to the changing marine environment.

## Introduction

Chitin, which is a polysaccharide composed of beta-1,4-linked N-acetylglucosamines (GlcNAc), is a tough leathery substance that constitutes the exoskeleton of arthropods and some mollusks, coelenterates, and protozoa (Zobell and Rittenberg, 1938; Tamadoni Jahromi and Barzkar, 2018). Chitin is the most abundant biomacromolecule in the marine environment (Itoh and Kimoto, 2019), and its annual natural production has been estimated to be 10^10^–10^12^ tons (Tharanathan and Kittur, 2003). Despite its obstinateness and abundance, the accumulation of chitin has not, to date, been detected in marine sediment (Keyhani, 1999), indicating a rapid turnover in seawater. As carbon and nitrogen sources, chitin is mostly digested by chitinolytic proteins produced by bacteria and fungi (Tharanathan and Kittur, 2003). To breakdown chitin, bacteria secret three types of chitinolytic protein, i.e., lytic polysaccharide monooxygenases (LPMOs, EC 1.14.99.53), chitinases (EC 3.2.1.14), and β-N-acetylheoxsaminidases (EC 3.2.1.52). LPMOs invade and loosen the chitin crystalline structure and produce even-numbered oligomers, and β-N-acetylheoxsaminidases yield GlcNAc from the non-reducing end sugar of oligosaccharides (Vaaje-Kolstad et al., 2013). Chitinases are indispensable for chitin degradation (Hayes et al., 2017), which release chitooligomers from the free chain ends. Additionally, chitinases have been grouped into four glycosyl hydrolase (GH) families based on amino acid sequences and structural dissimilarity, i.e., GH18, GH19, GH23, and GH48 (Lombard et al., 2014; Hayes et al., 2017).

Most bacterial chitinases belong to the GH18 family (Larsen et al., 2011), which has a (β/α)_8_ TIM-barrel enclosing the crucial and signature catalytic DXXDXDXE motif and a chitin-binding motif SXGG (Van Aalten et al., 2000). GH18 chitinases may be classified into three subfamilies based on sequence homology, i.e., ChiA, ChiB, and ChiC; all three subfamilies are multi-modular. Thus, ChiA has an N-terminal chitin binding module with a fibronectin III (FnIII) like fold (Perrakis et al., 1997). In comparison, ChiB has a C-terminal 5/12 CBM chitin binding domain (Van Aalten et al., 2000), whereas a C-terminal FnIII module coupled to a downstream 5/12 CBM chitin binding module occurs in ChiC (Payne et al., 2012). FnIII is a type of immunoglobulin-like (Ig-like) module, which is not directly responsible for chitin binding but directs the substrate to the catalytic groove (Perrakis et al., 1997). CBM5/12 is a chitin binding domain (ChtBD), which is vital in chitin binding and degradation (Watanabe et al., 1994). ChiA and ChiB are processive exo-chitinases, whereas ChiC is an endo-active non-processing chitinase. In ChiA and ChiB, the chitin-binding domains are situated in opposite directions, indicating these two enzymes work at different ends of the chitin chain. ChiA degrades chitin from the reducing end, whereas ChiB works at the non-reducing end (Hult et al., 2005).

Chitinolytic bacteria are ubiquitous and comprise a diverse range of species in the marine environment. Bacteria with chitin degradation ability are observed in different phyla, and include the genera *Vibrio* and *Pseudoalteromonas*, and *Serratia* within the phylum *Proteobacteria*; *Paenibacillus* and *Bacillus* within *Firmicutes*; and *Flavobacteria* within *Bacteroidetes* (Watanabe et al., 1990; Cottrell and Kirchman, 2000; Hunt et al., 2008; García-Fraga et al., 2015; Itoh and Kimoto, 2019). The genus *Vibrio*, which occurs widely in marine environments, is capable of responding quickly to environmental change, and blooming in certain conditions (Gomez-Gil et al., 2014; Zhang X. et al., 2018). Although they account for <1% of the total culturable and nonculturable bacterioplankton, *Vibrio* spp. comprised ~10% of cultured marine bacteria (Eilers et al., 2000). Their rapid reproduction and extensive substrate (chitin, alginate, and agar) utilizing capacities make vibrios vital in the marine carbon cycle, especially with regard to the chitin turnover (Zhang X. et al., 2018). The chitin utilization process of vibrios may be divided into four parts. At the initial stage of degradation, bacteria move toward the chitin source by chemotaxis. Then, vibrios adhere to the chitin surface and form biofilms (Yu et al., 1993). At the same time, regulators activate the chitin utilization system (Li and Roseman, 2004; Klancher et al., 2020). Next, the cells produce and secrete extracellular chitinolytic enzymes to break down the biopolymer into oligosaccharides, and finally uptake the products as nutrients (Itoh and Kimoto, 2019). The catabolic cascade of chitin utilization in vibrios involves many genes coworking with at least 3 transporters, 2 regulator systems, and a set of enzymes. To date, there is supporting experimental evidence with *V. cholerae* (Meibom et al., 2004; Kirn et al., 2005; Li et al., 2007; Blokesch, 2012; Hayes et al., 2017; Klancher et al., 2020), *V. furnissii* (Yu et al., 1993; Keyhani et al., 1996; Keyhani and Roseman, 1996) and *V. harveyi* (Montgomery and Kirchman, 1994; Svitil et al., 1997; Suginta et al., 2013).

Chitin utilization is a descendant feature of *Vibrio* spp. (Hunt et al., 2008), with chitinase-coding genes identified in their genomes. Bioinformatic analyses showed that 18 out of 20 *Vibrio* species harbored at least 5 chitinase coding genes (Lin et al., 2018). Ten different chitinolytic proteins were identified in *V. harveyi* when growing on different chitin and their analogs, but amino acid sequences of the enzymes are not available (Svitil et al., 1997). Several chitinases from *Vibrio* species have been purified for further study. Certainly, most research has focused on the characterization and enzymatic properties of chitinases (Supplementary Table S1). Also, one GH18 chitinase (Q9AMP1) from *V. harveyi* has been studied for its catalytic mechanism by site mutation and crystallization (Suginta et al., 2007; Pantoom et al., 2008; Songsiriritthigul et al., 2008; Suginta and Sritho, 2012). Although several copies of chitin-encoding genes may exist in a single *Vibrio* strain (Lin et al., 2018), only a few enzymes have been validated experimentally within the same strain. Different chitinases may be of importance and have been ranked by knock-out and corresponding complements in *V. cholerae* and *V. parahaemolyticus* (Hayes et al., 2017; Anusuya and Miyoshi, 2022). However, the co-working mechanism among various chitinases and their contribution to the adaptation of vibrios in changing environments remains unclear. Here, a chitinolytic strain *V. harveyi* WXL538 was recovered from estuarine water, and the complete genome sequence was determined, exploring its chitin degradation mechanism and potential adaptive strategies to variable environments. The putative chitinases were expressed heterologously, and the chitinolytic activity of the crude extract was examined. Active enzymes capable of degrading colloidal chitin were purified and characterized (Chi4668, Chi4733, Chi540). The disparity in their enzymatic properties could show light on the co-working of chitinases in chitin degradation and how they contribute to adaptation to changing environments.

## Materials and methods

### Strains, media, and growth conditions

*Vibrio harveyi* WXL538 was recovered from the East China Sea (122.56°E, 31.35°N) at a water depth of 25 m during the cruise of the R/V *Dong Fang Hong 2* in October 2015 (Liang et al., 2019). It was isolated on thiosulfate citrate bile salts sucrose (TCBS) agar (Hopebio, China), and showed chitinolytic capacity on colloidal chitin agar. Purified WXL538 was grown on marine agar 2216E (MA; Becton Dickinson) at 28°C. *Escherichia coli* (*E. coli*) BL21(DE3) was cultured on Luria-Bertani (LB) medium at 37°C with shaking at175 rpm/min and used for protein expression (Tang et al., 2015). The chitin degradation ability at 4, 16, 28, 37, and 50°C, pH 5, 6, 7, 8, 9, 10, and 11 were examined on MA supplemented with 1% colloidal chitin following incubation for 7 days. The examination of the effect of pH used the following buffer systems: MES (pH = 5.0 and 6.0), MOPS (pH = 7.0), Tricine (pH = 8.0), TAPS (pH = 9.0), and CAPS (pH = 10.0 and 11.0).

### Genome sequencing, annotation, and identification of WXL538

The genomic DNA of *V. harveyi* was extracted using the phenol-chloroform-isoamyl alcohol extraction protocol described by Marmur (1961), and the 16S rRNA genes were sequenced to achieve validation of the strain. The sequencing and assembling of total genomic DNA were the same as the methods of Lin et al. (2018). The complete genome sequences of *V. harveyi* WXL538 has been deposited in NCBI GenBank under the accession number CP045070 and CP045071. The genome sequences of other *Vibrio* strains were downloaded from GenBank. Coding sequences (CDSs) prediction and annotation were carried out using Rapid Annotations using Subsystems Technology (RAST) (Overbeek et al., 2014) and Prokka (Seemann, 2014). Functional prediction of CDSs was carried out by BLAST+ 2.2.24 (Altschul et al., 1997) searching against protein databases COG (Tatusov et al., 2000), KEGG (Kanehisa et al., 2004) and NR (Pruitt et al., 2007). Genes encoding CAZymes and peptidase were further annotated with dbCAN2 (Zhang H. et al., 2018) and MEROPS (Rawlings et al., 2018) v12.0 database, respectively. The taxonomic position of WXL538 was based on 16S rRNA gene phylogenetic Maximum-Likelihood tree construction by FastTree (Price et al., 2009), with 1,000 bootstraps. The genomes of other *Vibrio* species were downloaded from Genbank, and their 16S rRNA sequences were picked out from their Prokka annotation result. The taxonomic status of strain WXL538 was also checked with the calculations of DDH (DNA–DNA hybridization) and ANI (average nucleotide identity), which were carried out with GGDC (Meier-Kolthoff et al., 2013) and pyANI (Pritchard et al., 2016).

### Bioinformatic analysis of chitinases

Eight chitinase-coding genes were identified. Amino acid sequences of chitinase were analyzed by BLASTP against Swiss-Prot databases and PDB^1^ to obtain function and structure information. Multi-sequence alignment was carried out in muscle, and visualized by GeneDoc (Nicholas et al., 1997). The phylogenetic neighbor-joining tree of chitinase and related chitinase was constructed by MEGA version X (Kumar et al., 2018), with 1,000 bootstraps, and sequences of other chitinase were downloaded from Swiss-Prot and CAZy databases. The Molecular mass and pI of chitinases were predicted by the ExPASy database (Artimo et al., 2012). The conserved domains were predicted with the Simple Modular Architecture Research Tool (SMART) (Schultz et al., 2000) and the Conserved Domain Database (CDD) online service (Lu et al., 2020).

### Expression, purification, and activity detection of recombinant chitinases

To obtain recombinant proteins without signal peptides, the putative chitinase-coding gene was amplified with the primer pairs shown in Supplementary Table S2. The genomic DNA of *V. harveyi* WXL538 was used as a template for polymerase chain reaction (PCR) amplification of chitinase with Prime STAR®HS DNA polymerase. PCR products were purified using a gel extraction kit (Biomed, Beijing), then ligated into the pET-24a(+) expression vector. The recombinant vectors were transformed into *E. coli* BL21(DE3) for target protein expression. Positive clones were screened by PCR. The expression and purification of chitinases were carried out according to the approach of Tang et al. (2015), with modifications. The recombinant cells were grown in LB medium supplemented with kanamycin (50 μg mL^−1^) at 37°C until reaching the logarithmic phase (OD600 nm = 0.4–0.6). The recombinant protein was induced by the addition of 0.1 mM of IPTG at 16°C for 12 h. Cells were harvested by centrifugation at 8,000 g for 10 min and then washed with binding buffer (20 mM Tris, 50 mM NaCl, pH = 8.0). The suspensions were sonicated on ice, then centrifuged at 12,000 rpm for 10 min, and filtered through a 0.22-μm-pore-size filter to remove intact cells and debris. The sterile supernatant was loaded onto nickel-charged affinity resin (Ni-NTA, Qiagen). Proteins were purified according to the manufacturer’s recommendations. The purified recombinant chitinases were checked by 12% sodium dodecyl polyacrylamide gel electrophoresis (SDS-PAGE) conforming to the method of Laemmli (1970). Protein concentration was measured with the Bradford (1976) method using bovine serum albumin (BSA) as the standard.

In this study, 1% (w/v) colloidal chitin was the substrate of chitinase activity assays and prepared in accordance with the method of He et al. (2020) using shrimp chitin powder (Sigma-Aldrich). Chitinase activity was assayed based on the methods of Lee et al. (2007) with modification. Shortly, after the 30-min-incubation of 150 μL of the substrate at 50°C, 50 μL chitinase was added and continued incubating at 50°C for 1 h. The reducing sugars released were measured by the modified dinitrosalicylic acids (DNS) method (Miller, 1959). One unit (U) of chitinase activity was defined as the amount of enzyme that released 1 μmol of reducing sugars per minute per 1 mL protein under the assay conditions by using GlcNAc as the standard. For those chitinases which could not hydrolyze colloidal chitin, their activity was further tested with fluorescent substrates, MUF-β-D-N,N′-diacetylchitobiose [MUF-(GlcNAc)_2_] and MUF-β-D-N,N′,N″-triacetylchitobiose [MUF-(GlcNAc)_3_] (Sigma-Aldrich), according to the method of McCreath and Gooday (1992).

### Effect of various conditions on activity and stability of chitinases

To test the optimal temperature of chitinases, the reaction mixtures containing 1% chitin were incubated at 4, 10, 16, 28, 37, 45, 50, 60, and 70°C for 1 h, and then the reducing sugars released were measured by the modified dinitro salicylic acids (DNS) method described above. For thermostability, the enzyme samples were pre-incubated individually at 4, 10, 16, 28, 37, 45, 50, 60, and 70°C for 1 h, and then the residual activities were detected at the optimal temperature. For the optimal pH, the substrate for the assay was dissolved in different buffers with pH ranges from 2.0 to 11.0 (at intervals of 1.0): i.e., 0.05 M glycine-HCl (pH 2–5), 0.1 M citrate (pH 3–6), 0.05 M Na_2_HPO_4_-citrate (3–7), 0.05 M Tris–HCl (pH 7–9), 0.05 M glycine-NaOH (pH 10–11) and 0.05 M Na_2_HPO_4_-NaOH. For pH stability, the purified enzyme samples were pre-incubated in the previously mentioned buffers at 4°C for 1 h before the standard assay. Chitinase was incubated in the presence of K^+^, Ca^2+^, Mg^2+^, Al^3+^, Zn^2^, Cu^2+^, Co^2+^, Ba^2+^, Sr^2+^, Ni^2+^, and chemical reagents (SDS and urea) with concentrations of 1 mM and 10 mM for 1 h, and then the residual activities were monitored.

### Kinetic parameters and hydrolytic properties of chitinases

For kinetic parameter analyses, chitinases were incubated for 1 h at their optimal temperature with colloidal chitin in concentrations of 0.05, 0.1%, 0.2–0.8% (at intervals of 0.2%), 1.0, 1.25, and 1.5% (w/v), and determining the amount of reducing sugar by DNS methods. The Km and Vmax values were calculated following the Michaelis–Menten equation (Raaijmakers, 1987). Oligosaccharides produced from colloidal chitin, (GlcNAc)_3_ and (GlcNAc)_4_ by enzymatic reactions were analyzed by silica gel thin layer chromatography (TLC) using procedures described by He et al. (2020). Substrates at 1% (w/v) were fully and, respectively, mixed with purified chitinase, and then incubated at the optimal temperature for 5, 10, 15, 30, 45, and 60 min. After centrifugation, the supernatants were tested for chitinolytic activities.

## Results

### The genomic analysis and taxonomy of WXL538

The total genome size of *V. harveyi* WXL538 was 6.01 Mb, containing 2 circular chromosomes of unequal size (3.7 Mb and 2.3 Mb). The calculated C + G content was 44.86%. There were 5,688 CDS (coding sequences), 115 pseudogenes, 175 RNAs (fourteen 5S rRNAs, thirteen 16S rRNAs, twelve 23S rRNAs, 134 tRNAs, and 4 ncRNAs) and 1 CRISPR predicted in the genome. Among these predicted 5,688 CDS (coding sequences), 74.17% (4219) were found in COG categories, 53.48% (2969) matched in KEGG, and 86.88% (4942) of genes were also identified in the NR database. The COG functional annotation of the genome showed that genes related to transcription (K), amino acid transport and metabolism (E), and signal transduction mechanisms (T) were abundant in strain WXL538 (Supplementary Figure S1). According to KEGG annotation, except for poorly characterized protein, there are 94 metabolic pathways in WXL538’s genome (containing more than 2 genes), which contains 209 pathways and BRITE hierarchies. In the genome of WXL538, 47 two-component-system-related genes were identified. The genes that could sense the external stimuli and accordingly regulate the metabolic response are listed in Supplementary Table S3. Besides, WXL538 harbored a set of chemotaxis genes and nearly 50 motile coding genes (Supplementary Table S4).

To investigate the taxonomy of strain WXL538, an unrooted Maximum-Likelihood tree based on 16S rRNA gene sequences of *Vibrio* species was constructed. As shown in Supplementary Figure S2A, strain WXL538 was clustered into a branch with five other *V. harveyi* strains, which was further supported by DDH and ANI values (Supplementary Figure S2B). DDH and ANI were used to measure the genetic distance between genomes. DDH was regarded as the taxonomic gold standard for species delineation in *Archaea* and *Bacteria* with criteria of 70% (Meier-Kolthoff et al., 2013). ANI represents a mean of identity/similarity values between homologous genomic regions shared by two genomes, minimizing bias brought about by HGT events and variable evolutionary rates of different populations. The ANI delimitation criterion for different species was 95–96% (Lin et al., 2018). The DDH values of strain WXL538 with the other five *V. harveyi* strain ranged from 88.2 to 87.8%, higher than that with other *Vibrio* species (21.6–35.7%) and the 70% species delimitation criterion. The ANI values of strain WXL538 with five other *V. harveyi* strains were all 98.7%, higher than that with other *Vibrio* species (85.3–89.7%) and the 95% species delimitation criterion (Supplementary Figure S2B). Thus, WXL538 was identified as *V. harveyi*.

### Multi-carbon-source utilization potential of strain WXL538

To explore the utilization of carbon sources, we blasted the genome’s protein against MEROPS and CAZY databases, collections of peptidases, and Carbohydrate-Active enzymes (CAZyme), respectively. MEROPS identified 124 peptidases that could be categorized into eight families according to their amino acid sequence similarity. Among them, 46 were metallo peptidases and 39 were serine peptidases. More details are shown in Supplementary Figure S3. In terms of carbohydrate utilization, 113 identified proteins were divided into six enzyme classes, over half of which were glycoside hydrolases (GHs) and glycosyl transferases (GTs), and the remainder were carbohydrate esterases (CEs), polysaccharide lyases (PLs), carbohydrate-binding modules (CBMs) and auxiliary activities (AAs). These enzymes allow WXL538 to utilize more types of substances for nutrition. Based on the genome annotation, WXL538 has the potential to degrade alginate in a cascade as that one PL6 enzyme cut the polysaccharide into an oligosaccharide, which was the substrate of two PL17 enzymes (Hehemann et al., 2016). Furthermore, it harbors thirteen putative enzymes, which may degrade chitin, including six chitinases of GH18, a chitinase of GH19, an unclassified chitinase, three hexosaminidases of GH20 and two LPMOs of AA10. The classification of carbohydrate-active enzymes was based on the similarity of amino acid sequence and their common ancestor, so some enzymes of different substrates are classified into one CAZyme family (Lombard et al., 2014). Besides, some polysaccharides consisted of various monosaccharides and glycosidic bonds, and their degradation involved more than one kind of carbohydrate-active enzyme (Lombard et al., 2014). Thus, we could identify only a few specific substrates the strain may utilize.

### Chitin degrading ability and related genes in *Vibrio harveyi* WXL538

**Figure 1:**
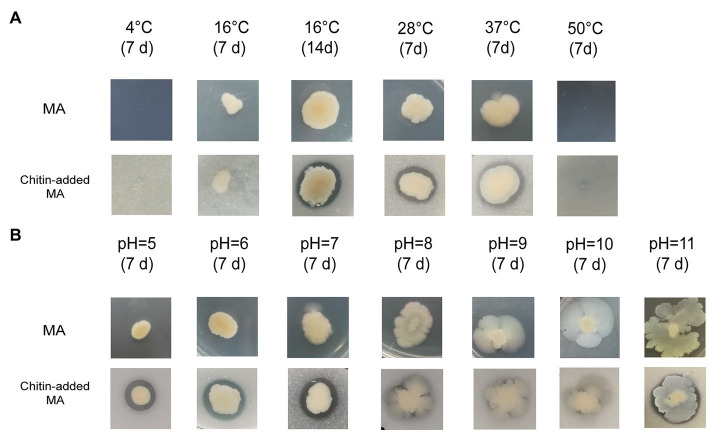
Growth and chitin degradation of *Vibrio harveyi* WXL538 under different conditions. **(A)** Conditions varied at temperature. **(B)** Conditions varied at pH.

**Figure 2:**
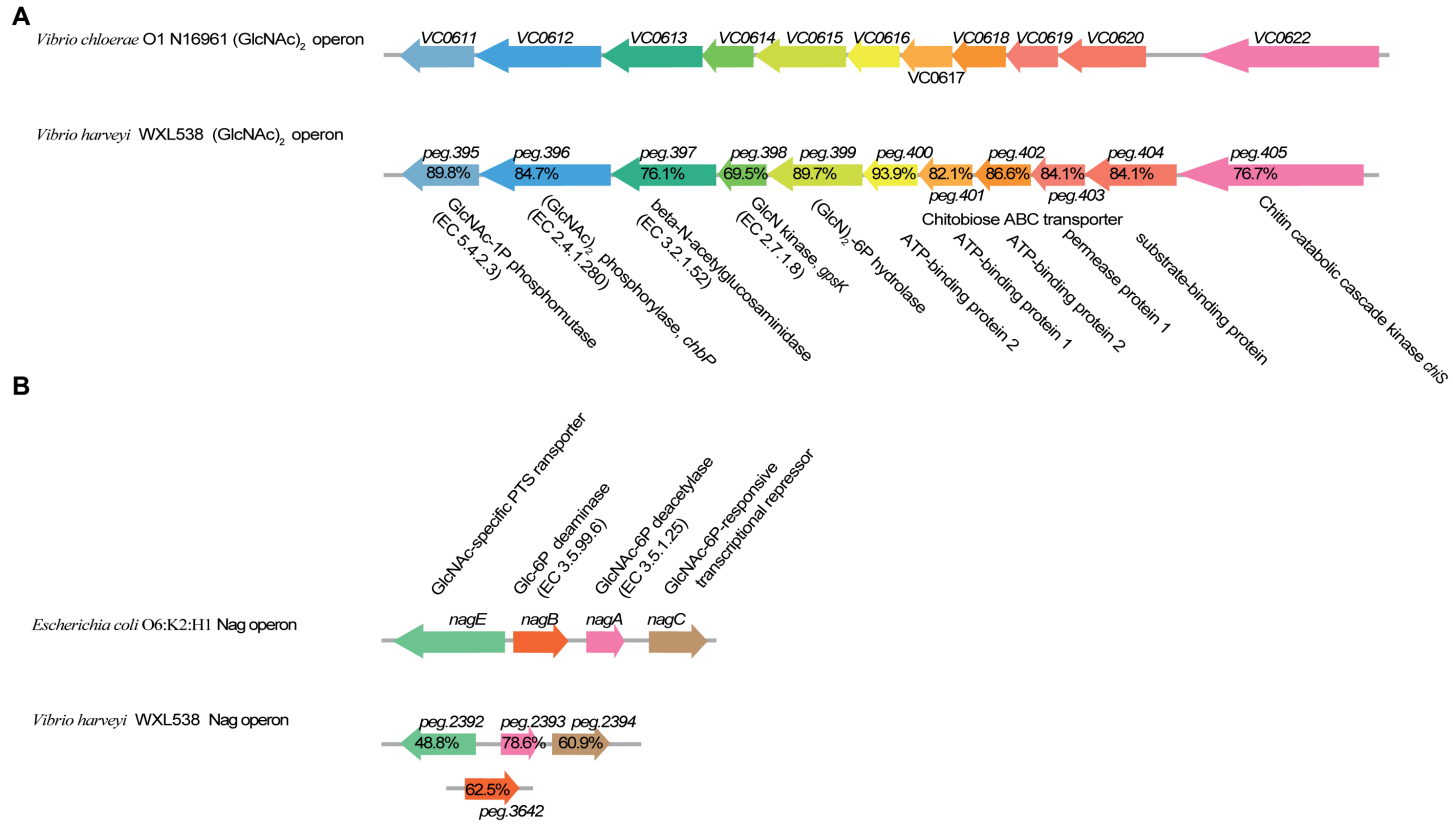
Comparison of (GlcNAc)_2_ operon and Nag operon in *Vibrio harveyi* WXL538 and *V. cholerae* O1 N16961. **(A)** (GlcNAc)_2_ operon. **(B)** Nag operon. The percentage referred to the identity of amino acid sequences.

WXL538 showed chitin degrading activity at a broad range of temperatures, i.e., 16°C, 28°C, and 37°C, and pH 5–11 (Figure 1). A whole set of chitin-metabolism-related genes had been identified in the genome of WXL538: eight putative chitinases (Chi4733, Chi540, Chi4668, Chi4963, Chi5174, Chi3480, Chi2497, and chitodextrinase Chi44930), two LPMOs which loosen the chitin crystalline structure and three beta-N-acetylglucosaminidases, which digest chitin into oligomers: a chitin-oligosaccharide-specific porin ChiP, which absorbs oligomers; and two downstream chitin degradation operons, (GlcNAc)_2_ operon and Nag operon. The two operons were both located on the larger chromosome and showed high similarity with its counterpart initially characterized in *V. cholerae* O1 and *E. coli* (Figure 2). The N, N′-diacetylchitobiose [(GlcNAc)_2_] operon, containing genes coding GlcNAc-1P phosphomutase, (GlcNAc)_2_ phosphorylase ChbP, glucosamine (GlcN) kinase GpsK, (GlcNAc)_2_ -specific ABC transporter and kinase ChiS (chitin metabolism regulator), formed a pathway to uptake and transform (GlcNAc)_2_ into available monomer. Nag operon included genes coding GlcNAc-specific PTS component NagE (the GlcNAc transporter), GlcNAc-6P-responsive transcriptional repressor NagC, GlcNAc-6P deacetylase NagA and GlcN-6P deaminase NagB. NagA and NagB, which transform the GlcNAc into fructose-6-P for energy metabolism, linking the chitin utilization to the EMP pathway. Unlike *E. coli* and *Photobacterium* species, of which the *nagB* gene was located between *nagE* and *nagA* (Giubergia et al., 2017), *nagB* of strain WXL538 was located on the other chromosome, far from the Nag operon. This phenomenon has also been reported in other *Vibrio* species, such as *V. coralliilyticus* S2052, *V. crassostrase*, *V. cyclitrophicus* FF75, and *V. parahaemolytics* AQ3810 (Giubergia et al., 2017).

**Figure 3:**
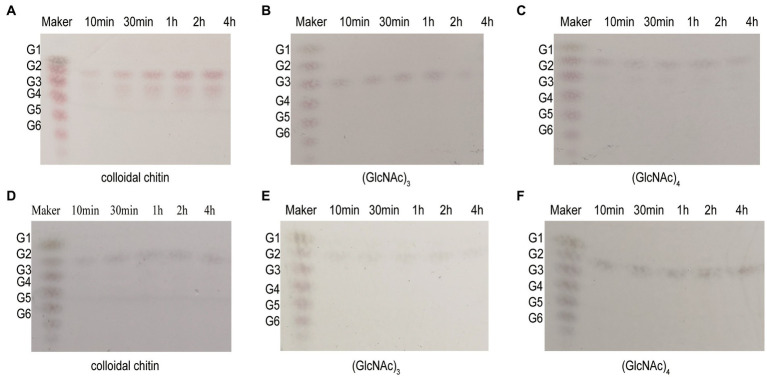
Neighbor-joining tree based on amino acid sequences of putative chitinases in WXL538 and other known chitinases. A hexosaminidase from GH20 was used as an outgroup. Bold Font indicated the chitinases were from *V. harveyi* WXL538. Numbers at nodes are the levels of bootstrap support (%). Scale bar, 0.2 substitutions per amino acid position.

**Figure 4:**
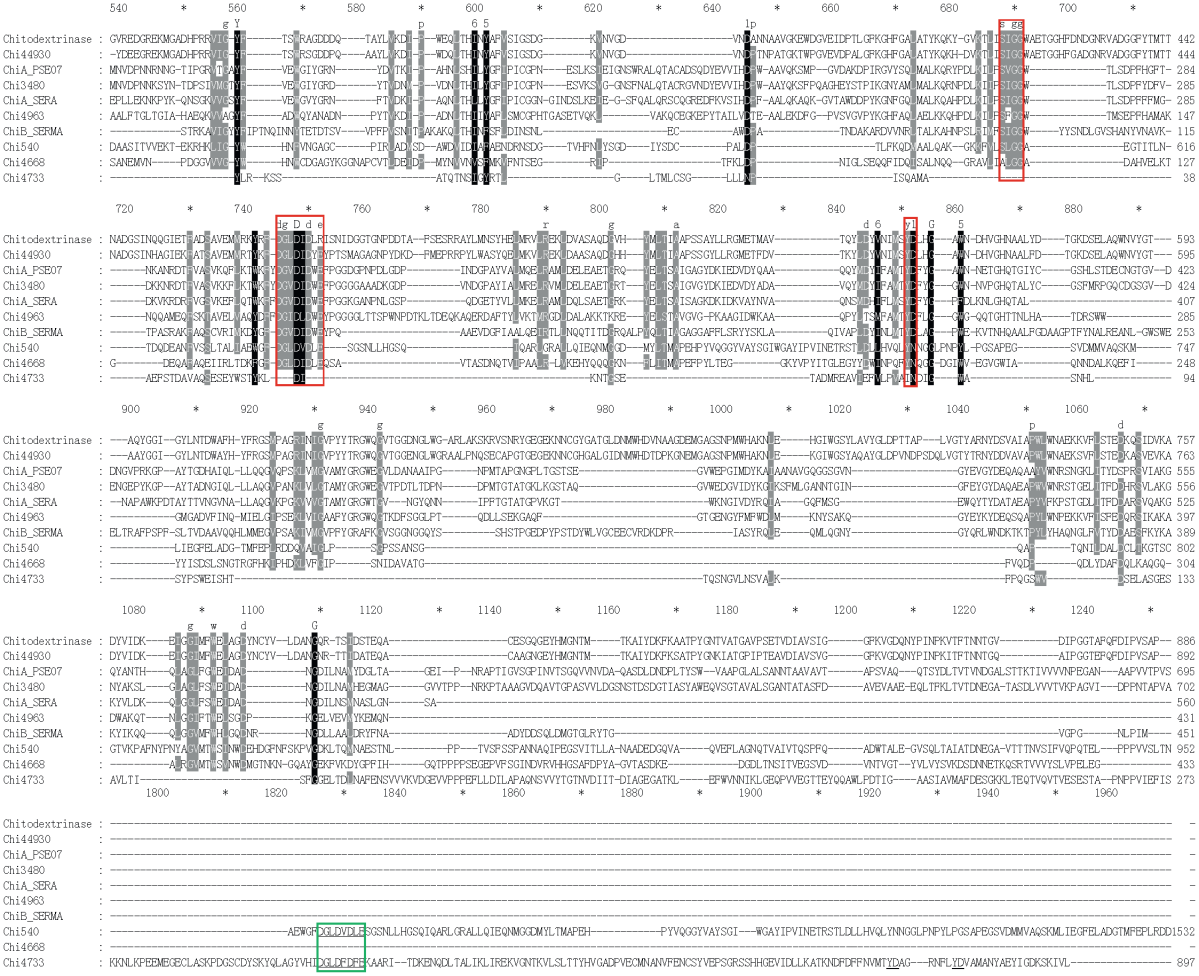
Multiple-sequence alignment of amino acid sequences of GH18 chitinases in strain WXL538 and referential experimentally valid chitinases from other species within the GH18 domain. The referential chitinases are 4TX8_A from *Chromobacterium violaceum*, MnChi460 from *Moritella marina* (4HMC), 122–588 aa of ChiA_PSEO7 (P32823) from *Pseudoalteromonas piscicida*, 309–784 aa of chitodextrinase (P96156) from *V. furnissii*, 160–544 aa of ChiA_SERA (P07254) and ChiB_SEMR (Q54276) from *Serratia marcescens*. Multiple-sequence alignment was performed by the MAFFT. Conserved amino acids are shaded in black (90% conservation or more) or in grey (70 to 90% conservation). Red boxes, three conserved domains of the GH18 family [SXGG, DXXDXDXE, and Y(D/N)].

In this study, we focused on the eight putative chitinases whose detailed information was summarized in Table 1. The predicted molecular weight of these chitinases varied between 40.00 to 130.82 kDa, and their pIs ranged from 3.71 to 5.18. Seven chitinases encoded a signal peptide at its N-terminal, while no signal peptides were found in Chi4733 (Supplementary Table S5). Other than Chi5174, the other seven chitinases fell into GH18 and GH19 (Figure 3). Chi2497 was GH19 chitinase with a catalytic domain and a chitin-binding domain. The rest six proteins belonged to the GH18 family and also contained a catalytic domain and one or two chitin-binding domains except Chi4963, which only possessed a single catalytic motif. Chi4733 and Chi540 had a chitin-binding domain at their N-terminal (ChiA): Chi4668 had a chitin-binding domain at C-terminal (ChiB), whereas Chi3480 and Chi44930 had binding domains at both sides (ChiC) (Supplementary Table S5). All of the six GH18 chitinases had three conserved motifs [SXGG, DXXDXDXE, Y(D/N)] (Figure 4). BLAST was performed using the amino acids sequence of Chi4733.

**Table 1:** Features of chitinases in *Vibrio harveyi* WXL538.

Chitinase	Coding nucleotide (bp)	Amino acid	Molecular Weight (kDa)	GH	pI	Chitinolytic substrate	Characterized or not	Most similar protein (organism, accession)	Accession	Cover-age (%)	Identity (%)
Chi4733	2,889	962	104.36	18	4.26	Colloidal chitin, (GlcNAc)_4_	Yes	Chain A, GH 9 (*Acetivibrio thermocellus* ATCC 27405)	P96156.1	23	22.18
Chi540	2,535	846	89.16	18	3.71	Colloidal chitin, MUF- (GlcNAc)_3_, (GlcNAc)_4_	Yes	Chain A, chitinase A (*Chromobacterium violaceum* ATCC 12472)	4TX8_A	37	52.01
Chi4668	1,680	561	61.15	18	4.37	Colloidal chitin, MUF- (GlcNAc)_3_, (GlcNAc)_4_	Yes	Chain A, Chitinase 60 (*Moritella marina*)	4HMC_A	90	61.01
Chi4963	1,290	431	48.00	18	5.18	MUF-(GlcNAc)_2–3_	Not	Chain A, Chitinase A (*Serratia marcescens*)	2WK2_A	94	33.65
Chi5174	3,618	1,207	130.82	None	4.66	Colloidal chitin, MUF- (GlcNAc)_3_, (GlcNAc)_4_	Not	Chain A, endoglucanase Z (*Dickeya dadantii 3,937*)	P96156.1	32	19.14
Chi2497	1,689	564	62.24	19	4.67	No activity be tested	Not	Chain A, chitinase (*Hevea brasiliensis*)	4MST_A	43	32.94
Chi3480	2,535	561	89.82	18	4.38	Failed in expression	Not	Chain A, Chitinase A (*Vibrio harveyi*)	3ARO_A	67	99.65
Chi44930	3,165	1,054	104.36	18	4.26	Failed in expression	Not	Chain A, chitinase A (*Chromobacterium violaceum* ATCC 12472)	4TX8_A	72	55.91

against the validated protein database (PDB, Swiss-Prot), the identities of matched chitinase sequences were lower than 23% within the coverage of 28%. Thus, we speculated that Chi4733 was a novel GH18 chitinase. Predicted with SMART, Pfam, and CDD, there was no known chitinolytic conserved domain but some carbohydrate-binding domains, such as an immunoglobulin-like (Ig-like) domain, chitin-binding domain type III (ChtBD3) and carbohydrate-binding modules (CBMs) in Chi5174 (Supplementary Figure S4; Supplementary Table S5).

### Heterologous expression, purification, and chitinolytic activity assay of chitinase

Among those eight chitinolytic-enzyme-coding genes, six had been successfully cloned and heterologously expressed in *E. coli* BL21 (DE3). The molecular masses of the purified proteins were estimated by SDS-PAGE, which were consistent with the predicted molecular masses (Supplementary Figures S5A–C). Activity assays were carried out with crude enzyme products. Thus, Chi4733, Chi540, Chi4668, and Chi5174 could hydrolyze colloidal chitin. Chitinase, which could degrade colloidal chitin, was able to degrade MUF-(GlcNAc)_3_ except Chi4733. Chi4963 lacked the Ig-like domain and could not hydrolyze the colloidal chitin but could hydrolyze both MUF-(GlcNAc)_2_ and MUF-(GlcNAc)_3_. No activity was tested in Chi2497. Chi4733, Chi540, and Chi4668 were successfully purified (Supplementary Figures S5D–F) and characterized (Table 2). The enzymatic properties of Chi4668 were described in our previous study (He et al., 2020). In the present study, we only describe the characterization of Chi4733 and Chi540.

**Table 2:** The enzymology properties of Chi4733, Chi540, and Chi4668.

Enzymology properties	Chi4733-Ni	Chi540-Ni	Chi4668-Ni
Total enzymatic activity (U)	3.51	5.38	3.16
Total protein content (mg mL^−1^)	0.02	0.04	0.08
Specific activity (U mg^−1^)	175.5	134.5	39.5
The optimal temperature (°C)	50	60	50
The optimal pH	4–6	6–8	3–6
Stable temperature (°C)	0–45	0–50	0–45
Stable pH	5–7	5–8	3–11
Hydrolytic products of colloidal chitin	(GlcNAc)_2_ and (GlcNAc)_3_	(GlcNAc)_2_	mainly (GlcNAc)_2_, with little (GlcNAc)_3_, (GlcNAc)_4_ and GlcNAc
The kinetic parameters:			
*V*max (mg U^−1^)	54.9	14.7	6.21
*K*m (mg mL^−1^)	2.1	0.48	2.75
*K*cat (s^−1^)	21.1	4.09	5.18
*K*cat/*K*m (s^−1^ M^−1^)	0.4	0.12	1.88

The specific activity of recombinant chitinases was observed when using colloidal chitin as a substrate. The total enzymatic activity of Chi4733 and Chi540 was individually 3.51 U and 5.38 U, the total protein content was 0.02 mg mL^−1^ and 0.04 mg mL^−1^, and the specific activity was 175.5 U mg^−1^ and 134.5 U mg^−1^.

### Effect of various conditions on the activity and stability of Chi4733 and Chi540

**Figure 5:**
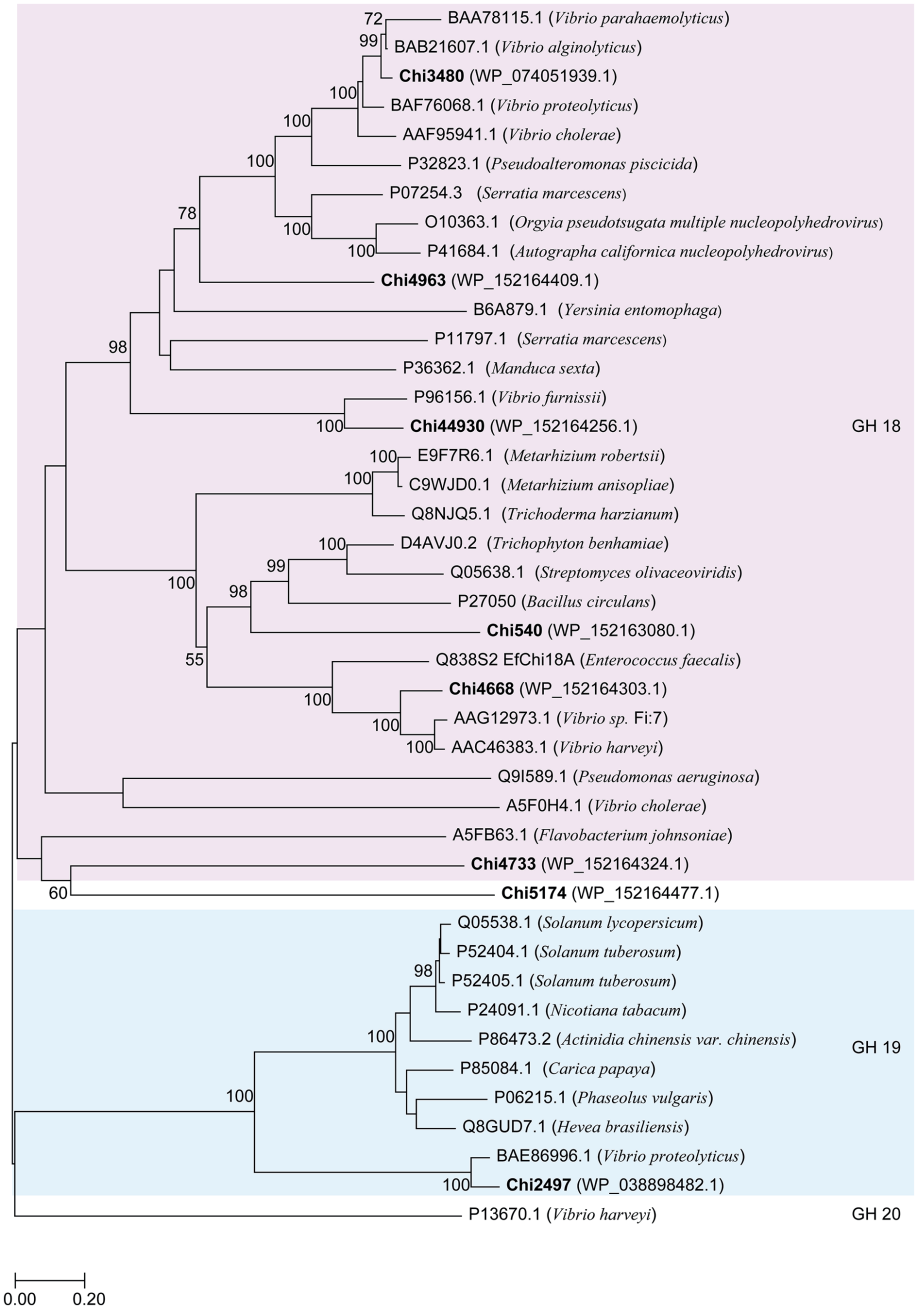
Effect of temperature and pH on Chi4733 and Chi540. **(A)** Optimal temperature and thermal stability of Chi4733. **(B)** Optimal temperature and thermal stability of Chi540. **(C)** Optimal pH of Chi4733. **(D)** pH stability of Chi4733. **(E)** Optimal pH of Chi540. **(F)** pH stability of Chi540.

Chi4733 showed optimal activity at 50°C. The activity of Chi4733 increased with temperature until peaking at 50°C and then dropping abruptly. When at 60°C, Chi4733 lost almost all its enzymatic activity. Regarding thermal stability, Chi4733 retained over 90% of its initial activity after 1 h of incubation at 0–45°C, but only 10% at 50°C (Figure 5A). The optimal temperature of Chi540 was 60°C, with inactivation at 70°C. Chi540 retained over 90% enzymatic activity after 1 h of incubation at 0–50°C and lost ~90% activity when incubated at 60°C for an hour (Figure 5B). The disparity of optimal temperature chitin degradation of strain growth on the chitin plate (37°C) and chitinase in the liquid reaction mixture (50–60°C) may result from the *in vivo* and *in vitro* chitinolytic process, and the different amino acid sequence features of each chitinase.

Chi4733 showed the optimum pH at 4.0–6.0 in different buffers (>80%). It showed relatively lower activity at pH 3.0 (~70%), pH 7.0 (~60%), and pH 8.0 (~60%), but lost almost all its activity at pH 2.0 and in alkaline environments (pH 9.0–12.0) (Figure 5C). For pH stability, Chi4733 retained over 80% of activity when incubated at pH 6–7 for 1 h, and maintained relatively lower activity at pH 3.0–5.0 (20 ~ 50%) and pH 8.0–12.0 (~10%). However, Chi4733 was deactivated in 0.05 M citrate buffer, pH 2.0 (Figure 5D). Chi540 showed an optimum pH of 5.0–8.0 in different buffers (>90%). Chi540 showed relatively lower activity at pH 4.0 (~70%) and pH 9.0 (~50%), but lost almost all its activity at pH 2.0 and pH 10.0–12.0 (<10%) (Figure 5E). For pH stability, Chi540 retained over 70% activity after an hour incubation in 0.05 M Na_2_HPO_4_-Citrate buffer at pH 6.0–8.0, but relatively lower activity at pH 3.0–5.0 (30 ~ 50%) and pH 9.0–12.0 (~10%) (Figure 5F).

The effects of metal ions and chemical reagents (EDTA, SDS, and urea) on enzymatic activity were examined (Supplementary Figure S6). Thus, Chi4733 was activated by Ca^2+^, Co^2+^, Sr^2+^ (10 mM), and Mg^2+^ (10 mM), but inhibited by Al^3+^ (10 mM), Zn^2+^, Cu^2+^, Ni^2+^ and SDS. Chi540 was activated by Sr^2+^ (10 mM), Ca^2+^ (10 mM), and Mg^2+^ (10 mM) but inhibited by Al^3+^, Zn^2+^, Cu^2+^, Ni^2+^, Ba^2+^, SDS, Sr^2+^ (1 mM), K^+^ (1 mM), and urea (10 mM).

### Kinetic parameters and hydrolysis properties of recombinant chitinases

**Figure 6:**
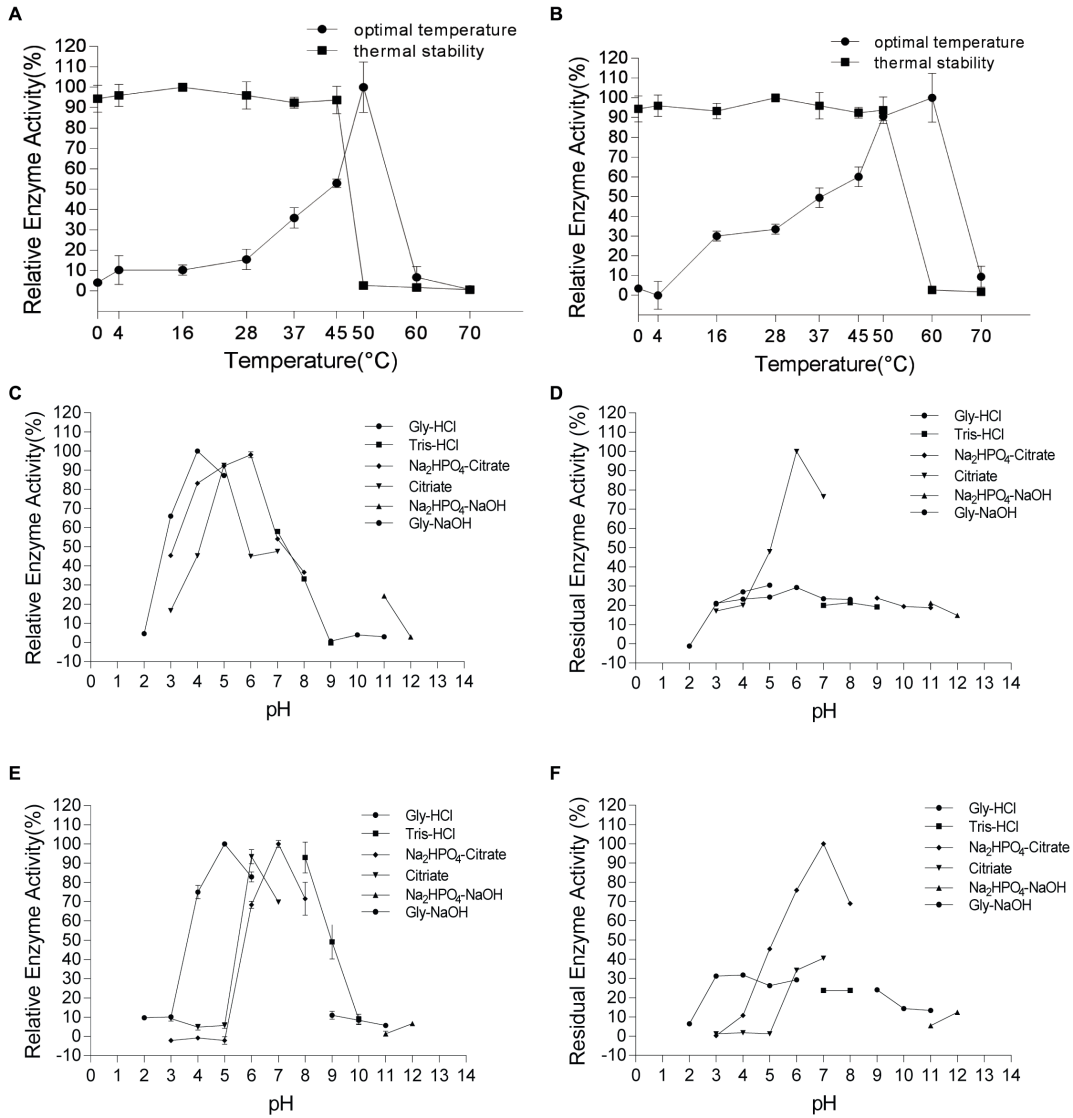
Hydrolysis property of Chi4733 and Chi540. **(A–C)** The degradation products of Chi4733 for 1% (w/v) colloidal chitin (GlcNAc)_3_ and (GlcNAc)_4_. **(D–F)** The degradation products of Chi540 for 1% (w/v) colloidal chitin, (GlcNAc)_3_ and (GlcNAc)_4_. Purified Chi4733 and substrates were incubated at 50°C, and Chi540 were at 60°C for different time intervals.

The Michaelis–Menten constant (*K*m) values of Chi4733 and Chi540 for colloidal chitin were 2.1 mg mL^−1^ and 0.48 mg mL^−1^, respectively. The *K*cat values of Chi4733 and Chi540 for colloidal chitin were 1.88 s^−1^ M^−1^ and 4.09 s^−1^ M^−1^, respectively (Table 2). The hydrolysis properties of recombinant chitinases on colloidal chitin and *N*-acetyl (GlcNAc)_3–4_ were investigated in detail. Thus, for the degradation of Chi4733, colloidal chitin was hydrolyzed into (GlcNAc)_2_, and (GlcNAc)_4_ was hydrolyzed into (GlcNAc)_2_, suggesting that Chi4733 was an endo-chitinase. In contrast, Chi540 hydrolyzed colloidal chitin into (GlcNAc)_2_ and (GlcNAc)_3_, (GlcNAc)_3_ into (GlcNAc)_2_ with GlcNAc, and (GlcNAc)_4_ into (GlcNAc)_2_, indicating that Chi540 was an exo-chitinase (Figure 6).

## Discussion

Chitin utilization is an ancestral feature of *Vibrio* species, which are ubiquitous and cultivatable members of the coastal bacterial community (Hunt et al., 2008; Giubergia et al., 2017; Lin et al., 2018; Zhang X. et al., 2018). Vibrios produce multiple chitinases to degrade chitin, and these exert an important role in environmental adaptation (Hayes et al., 2017; Adams et al., 2019; Klancher et al., 2020). Here, we sequenced and analyzed the genome of a chitinolytic strain, *V. harveyi* WXL538, and identified the complete pathway of chitin degradation, including the presence of multiple chitinases and the other genes involved in environmental adaptation. The validation and characterization of chitinases indicated that their different properties may enhance the chitin degradation ability of *V. harveyi* WXL538 in variable marine environments.

### Chitin metabolism and chitinases of *Vibrio harveyi* WXL538

The polysaccharide chitin is the most abundant biomolecule around vibrios, and this is of great significance for survival in the marine environment (Le Roux and Blokesch, 2018). The genome analysis of *V. harveyi* WXL538 showed that it harbors a complete set of chitin-utilized genes, which are conserved in *Vibrio* species (Meibom et al., 2004; Hunt et al., 2008). These genes include Nag and (GlcNAc)_2_ operons (Giubergia et al., 2017). Also, WXL538 contains eight putative chitinase encoding genes. Chitinase is an important pioneer in chitin degradation, cutting the macromolecule into soluble oligomers for further digestion. Certainly, many marine bacteria can produce chitinase, and include *Alteromonas* sp. strain O-7 (Orikoshi et al., 2005) and *Aeromonas salmonicida* SWSY-1.411 (Pentekhina et al., 2020), which has at least four chitinases; *Pseudoalteromonas* S91 (Techkarnjanaruk and Goodman, 1999) and *Serratia marcescens* 2,170 (Suzuki et al., 2002) contain three chitinases, respectively. In comparison, *V. harveyi* has more copies of chitinase. In vibrios, the number of chitinase coding genes varies from 0 to 7: 0 in *V. tritonius* JCM16456, 3 in *V. rotiferianus* B64D1, 6 in *V. cholerae* O1 N16961, and 7 in *V. nigripulchritudo* SFn1 (Hayes et al., 2017; Lin et al., 2018). However, they are regarded as putative chitinases due to the lack of conclusive experimental evidence. Within WXL538, five putative chitinases were active, i.e., their coding genes were not pseudogenes. More copies of active chitinase may enhance the chitinolytic ability of *V. harveyi*.

In *Serratia marcescens* 2,170, three chitinases of different enzymatic properties worked synergistically in chitin degradation (Suzuki et al., 2002). In terms of the chitin-degradation process of WXL538, endo-chitinase Chi4668, and Chi4733 cut the long chain into oligomers, and exo-chitinase Chi540 subsequently degraded them into (GlcNAc)_2_. The presumed cytoplasmic chitinase Chi4963 hydrolyzed the dimer into monomers. These chitinases form a possible chitin degradation cascade. Unlike other chitin-degrade microorganisms, vibrios encode a specific porin (ChiP) to import long chitin oligosaccharides into the periplasm (Chumjan et al., 2015), whereas others, for example, *Streptomyces,* directly absorb GlcNAc and (GlcNAc)_2_ (Itoh and Kimoto, 2019). The long oligomers in the periplasm may serve as signals to upregulate the chitin utilization regulon (Keyhani and Roseman, 1996; Li and Roseman, 2004). The extra step of chitin digestion not only allows it to obtain more nutrients (Suginta et al., 2013) but also activates chitin-degrading catabolism. According to Hayes et al. (2017), the importance of each chitinase in chitin-utilization was assayed through different chitinase-lacking mutants and corresponding ectopic expression in the mutant of *V. cholerae*, which harbored seven putative chitinase-coding genes, the results of which showed that different chitinases functioned differently in chitin degradation, i.e., ChiA2 was indispensable.

Chi4733 and Chi540 demonstrated great higher specific activity over 130 U mg^−1^, compared to that of other marine bacterial sourced chitinases and *Vibrio*-derived ones, e.g., *Pb*Chi70 from *Paenibacillus barengoltzii*, 30.3 U mg^−1^; chitinases from *Alcaligenes faecalis* AU02, 81.52 U mg^−1^; chitinase from *Micrococcus* sp. AG84, 93.02 U mg^−1^; chitinase from *Vibrio* sp. 11,211, 36.5 U mg^−1^; Chi1557 from *V. rotiferianus* WXL191, 23.42 U mg^−1^ (Tamadoni Jahromi and Barzkar, 2018; He et al., 2020). Chi4733 has low similarity with validated proteins in the PDB and Swiss-Prot database (identities were lower than 23% within the coverage of 23%), and there was no obvious difference between Chi4733 and the other GH18 chitinase in the GH18 domain (Figure 4), the strong activity of Chi4733 may come from an unknown binding or catalytic mechanism of chitinase. Chi4733 contains three Ig-like domains (269–338 aa, 432–501 aa, 527–593 aa), one ChtBD3 domain (366–417 aa, between the second and third Ig-like domain), and a GH18 domain (626–945 aa). We expressed the truncated Chi4733 with deletion of amino acids before Ig-like domains (232–962 aa), deletion of two Ig-like domains, and ChtBD3 domain (495–962 aa) or only GH18 domain (626–962 aa), and no colloidal chitin-hydrolyzing activity was detected in these truncated proteins. ChtBD and Ig-like domains both take part in chitin binding and directing the substrate to the catalytic groove, which is vital for colloidal chitin degradation (Watanabe et al., 1994; Van Aalten et al., 2000). The ChtBD domain is indispensable in chitin binding, but the Ig-like domain is not so. The loss of the ChtBD domain in chitinases largely impaired the colloidal chitinolytic and chitin-binding activities, whereas that of Ig-like domains in chitinases only significantly decreased their colloidal chitin-hydrolyzing activity, but did not affect their affinity to chitin (Watanabe et al., 1994). Interestingly, truncated protein retaining all predicted domains was inactive in colloid chitin degradation. We considered that 1–232 amino acids part may play an important role in Chi4733 chitin degradation, which needs further study. Though Chi5174 fell belongs to the GH18 chitinases in the phylogenetic tree (Figure 3), no known chitinolytic conserved domain was predicted with SMART, Pfam, and CDD (Supplementary Figure S4; Supplementary Table S5). Thus, we suspected that Chi5174 was a novel chitinase adopting a novel catalytic mechanism. These chitinases provided the novel potential for chitinase discovery and new materials for chitinase mechanism understanding.

### Adaptation strategies of strain WXL538 to the marine environment

Chitinase not only plays a crucial role in nutrient supplement by hydrolysis of chitin but may also take part in other life processes, such as horizontal gene transfer. A low level of (GlcNAc)_2_, the product of chitin degradation, may induce natural competence in *V. cholerae* (Meibom et al., 2005; Blokesch, 2012). In this study, the three characterized chitinases, Chi4668, Chi4733, and Chi540, showed stability under a wide range of temperatures and pHs, allowing the constant chitin-oligosaccharides supply for the strain to grow under adverse conditions. According to John and PAN (Reichelt and Baumann, 1973; Xiao-yi et al., 2005), *V. harveyi* may grow normally at 25–35°C, slowly at 45°C, but cannot grow under 50°C and 4°C. It was found that chitin may help *V. vulnificus* resist temperature stress to survive at 20°C, a relatively cold condition (Motes et al., 1998). In our study, *V. harveyi* could grow at 16–37°C in the culture medium adding 1% colloidal chitin, whereas it could not grow at 4°C and 50°C (Figure 1). Besides temperature, pH is an important factor affecting *Vibrio* spp. to adapt to the marine environment (Takemura et al., 2014). It has been reported that *V. harveyi* could grow normally within pH 5 to 9 and very slowly at pH over 10 (Xiao-yi et al., 2005). However, our results showed that *V. harveyi* may still grow under pH 10 and 11 (Figure 1). The chitin-adding culture makes *V. harveyi* grow faster in an alkaline environment. Moreover, Carla et al. (2008) found that chitin could ensure *V. cholerae* survive under acid stress; Also, Nalin et al. (1979) suggested that chitin could enhance the tolerance of *V. cholerae* to low pH and alum/chlorine. Like other chitinases from *vibrio* [i.e., *V. proteolyticus* (Itoi et al., 2007), *V. alginolyticus* H-8 (Ohishi et al., 1996) and *V. furnissii* (Keyhani and Roseman, 1996)], Chi4733, Chi540 and Chi4668 keep stable in the acid environment. Thus, chitin and chitinases help *V. harveyi* to resist extreme physiological conditions in the marine environment. Additionally, other than a whole set of chitin-degradation-related genes, WXL538 also harbored the potential of alginate utilization with the identification of one PL6 and two PL7 enzymes (Hehemann et al., 2016). Also, WXL538 harbored over 200 carbon utilization-related enzymes, indicating it could utilize a wide range of substances for nutrition, elevating its survival competence in changing marine environments.

The advantage of unicellular organisms to survive, grow, and compete with other microorganisms in changing environments is sensing and adapting to changes by modifying their cellular physiological metabolism (Miller et al., 2009). Bacteria may detect various environmental stimuli, including osmolarity, pH, temperature, salinity, and chemical ligands of diverse physicochemical properties. Strain WXL538 could respond to multi stimuli. Two-component systems (TCSs) are sets of proteins serving as a primary stimulus–response coupling mechanism to allow organisms to sense and respond to changes in many different environmental conditions (Stock et al., 2000). In the genome sequence of strain WXL538, 47 TCSs-related genes were identified, including *cusRS* and *cueR* (Montgomery and Kirchman, 1994), *envZ* and *ompR* (Siryaporn and Goulian, 2008), *arcAB* (Loui et al., 2009) and *narP, narQ, narL* (Rabin and Stewart, 1993), which respond to heavy metal, osmolarity, reactive oxygen stress and nitrate/nitrite stimulus, respectively. Those genes are the sensors and regulators of corresponding metabolisms, adjusting their physiology to remain alive. Motile bacteria with chemotaxis can swim and navigate themselves in the surrounding environment confers them with a competitive advantage in that it allows the cells to occupy and maintain niches that are optimum for survival and growth (Miller et al., 2009). In the genome sequence of WXL538, a complete set of genes encoding chemotaxis cascade were identified, including *cheW, cheV, cheA, cheB, cheY,* and *cheR*, allowing it to respond to attractants and repellents. Notably, 29 sensing proteins, i.e., methyl-accepting chemotaxis proteins (MCPs), were identified in strain WXL538, vastly more than *Escherichia coli* with only 5 MCPs, but similar to *Shewanella oneidensis* (an aquatic bacteria) with 27 MCPs (Miller et al., 2009). Additionally, 46 genes related to flagellar assembly were also found. The flagellum is the controller of the locomotion appendix (Colin and Sourjik, 2017) of motile bacteria, which is highly energy-consuming and requires a large number of genes to strictly control its synthesis and function (Le Roux and Blokesch, 2018). Besides, a large number of peptidases and Carbohydrate-Active enzymes allow strain WXL538 to utilize more types of substances for nutrition, enhancing the competence of strain in the marine environment.

## Conclusion

We studied the effects of different environmental factors on the chitin metabolism of *V. harveyi* WXL538, speculating that it exerts an important role in the adaptation of vibrios to marine life. WXL538 encodes eight putative chitinase and other genes involved in the complete degradation of chitin. Five chitinases (i.e., Chi4668, Chi4733, Chi540, Chi4963, and Chi5174) were expressed and validated. Chi4733, Chi540, and Chi4668 were purified, and revealed activity at wide temperature and pH ranges. According to differences in enzymatic properties and structure of chitinases, their different roles in chitin degradation were speculated. Validation of Chi4733 and Chi5174 provided a new understanding of the chitinase catalytic mechanism and expanded the potential of chitinase discovery. Overall, genomic analysis and the characterization of chitinase in *V. harveyi* WXL538 provided a better understanding of its adaptation to the changing marine environment. For Chi5174, no known chitinolytic domain has been identified, which should be the focus of further studies, including protein crystal structure analysis.

## Data availability statement

The original contributions presented in the study are included in the article/Supplementary material, further inquiries can be directed to the corresponding author.

## Author contributions

X-HZ: conceptualization, resources, supervision, and funding acquisition. LR and XW: data curation, formal analysis, and validation. LR, XH, YW, and PZ: investigation. LR, XW, YW, and X-HZ: methodology. LR and X-HZ: project administration. LR: visualization and writing—original draft. XW, RG, and X-HZ: writing—review and editing. All authors have read and agreed to the published version of the manuscript.

## Funding

This work was funded by the National Natural Science Foundation of China (41730530 and 92251303), the Fundamental Research Funds for the Central Universities (202172002), the Scientific and Technological Innovation Project of Laoshan Laboratory (2022QNLM030004-3, LSKJ202203201, and LSKJ202203206), and the Qingdao Postdoctoral Program (QDBSH20220202122).

## Conflict of interest

The authors declare that the research was conducted in the absence of any commercial or financial relationships that could be construed as a potential conflict of interest.

## Publisher’s note

All claims expressed in this article are solely those of the authors and do not necessarily represent those of their affiliated organizations, or those of the publisher, the editors and the reviewers. Any product that may be evaluated in this article, or claim that may be made by its manufacturer, is not guaranteed or endorsed by the publisher.

## Footnotes

^1^https://blast.ncbi.nlm.nih.gov/
