# Supplementary material for: Genomic analysis and chitinase characterization of Vibrio harveyi WXL538: insight into its adaptation to the marine environment
Source: Front Microbiol. 2023 Jul 3;14:1121720. doi: 10.3389/fmicb.2023.1121720 (PMC10350509; doi:10.3389/fmicb.2023.1121720)
Supplement: Supplementary file 1 [file Data_Sheet_1.PDF]

Genomic analysis and chitinase characterization of *Vibrio harveyi* WXL538: insight into its adaptation to marine environment.

Lingman Ran[1 †], Xiaolei Wang[1 †], Xinxin He[1], Ruihong Guo[1], Yanhong Wu[1], Pingping Zhang[1] and Xiao-Hua Zhang[1,2,3*]

[1]Frontiers Science Center for Deep Ocean Multispheres and Earth System, and College of Marine Life Sciences, Ocean University of China, Qingdao 266003, China

[2]Laboratory for Marine Ecology and Environmental Science, Laoshan Laboratory, Qingdao 266237, China

[3]Institute of Evolution & Marine Biodiversity, Ocean University of China, Qingdao 266003, China

† These authors contributed equally to this work.

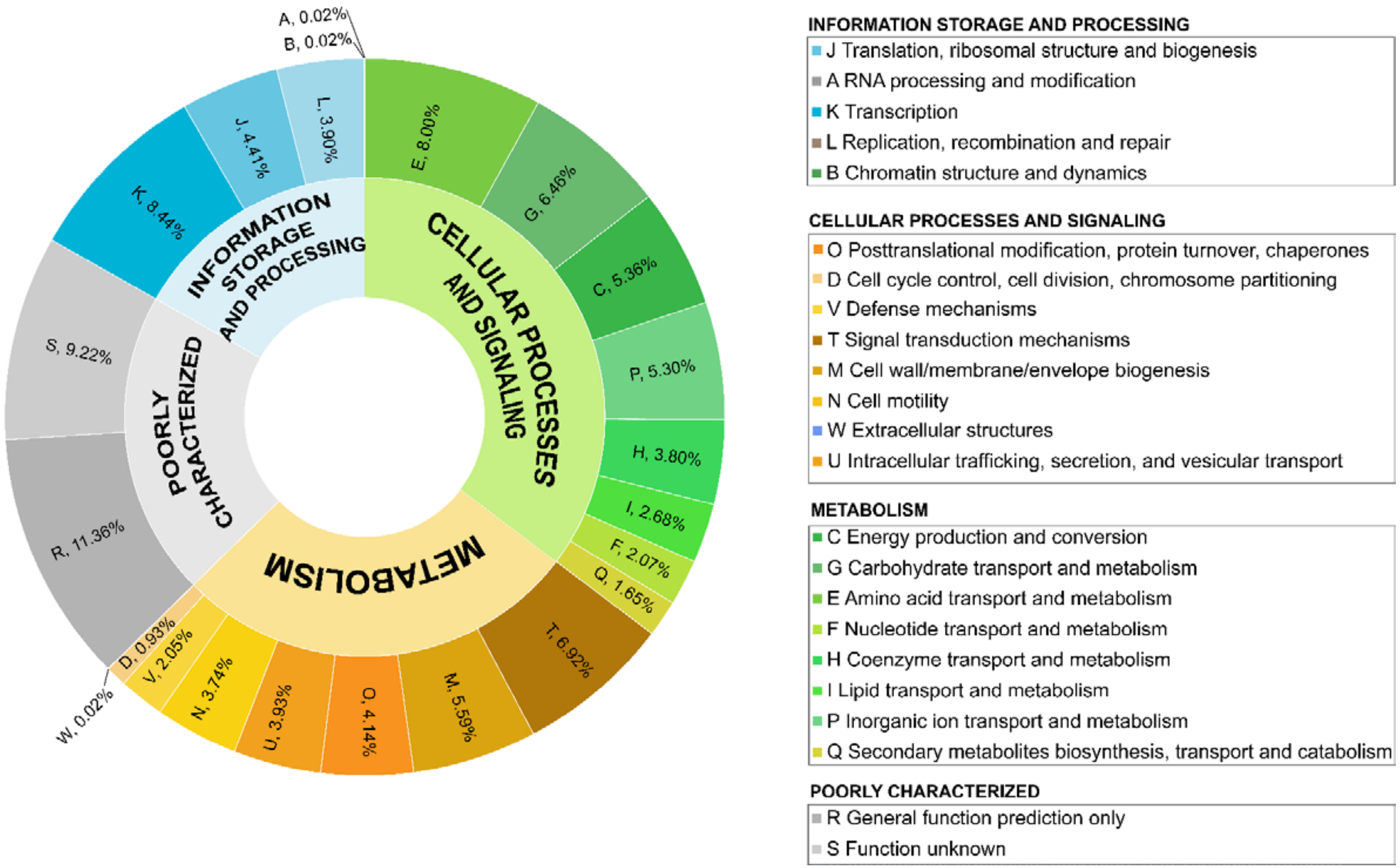

Figure.S1 Functional classification of CDSs encoded by WXL538 genome based on the COG. In total, 4,219 CDSs with orthologs in the COG database were classified and the percentages indicate the frequencies of CDSs with assigned functions.

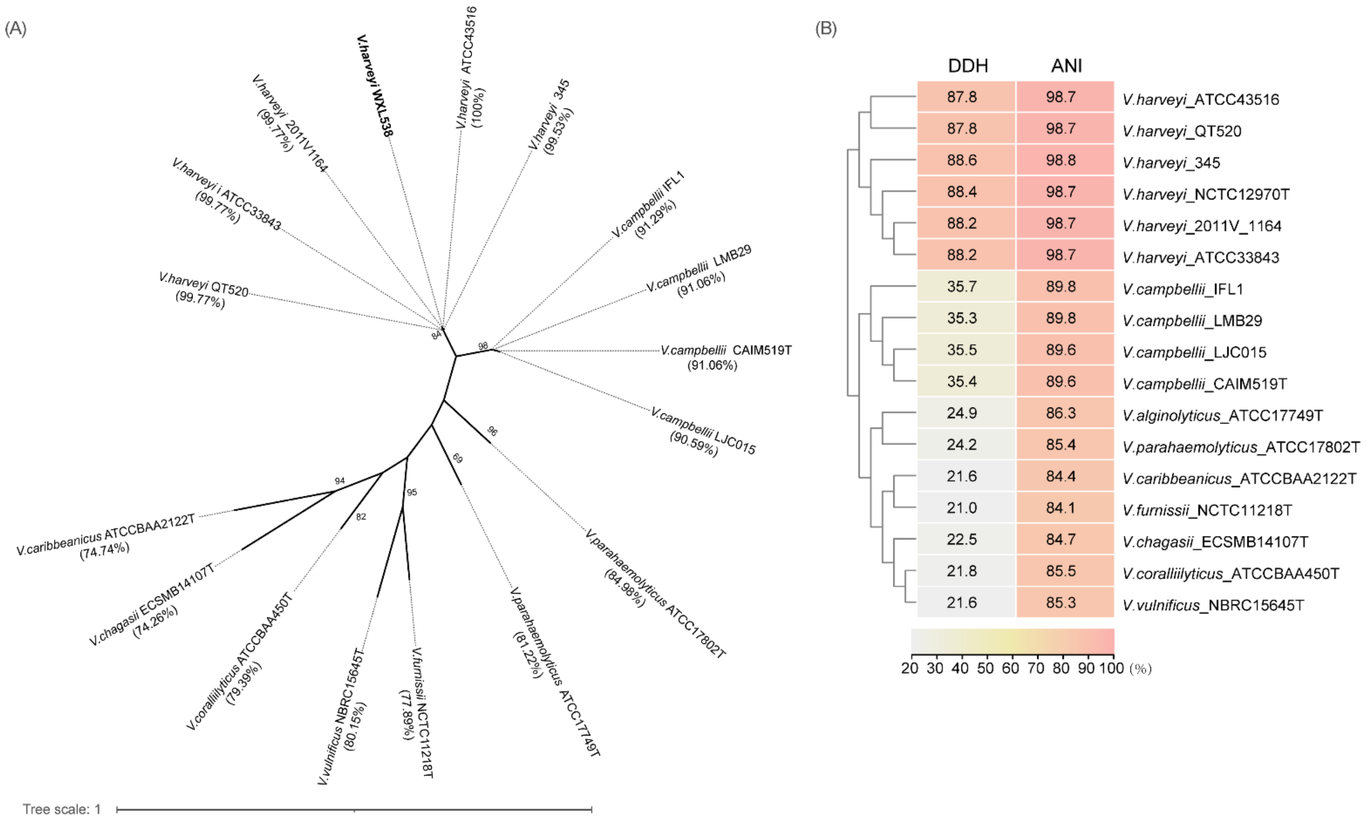

Figure S2. The taxonomic identification of strain WXL538. A, unrooted maximum-Likelihood tree based on 16S rDNA sequences of vibrio species. T, type strain. Percentage number, identity compared with 16S rDNA sequence of WXL538 strain. B, the DNA-DNA hybridization (DDH) and average nucleotide identity (ANI) values of strain WXL538 compared with other *Vibrio* species, which species delimitation criteria was 70% and 95%, respectively. T, type strain.

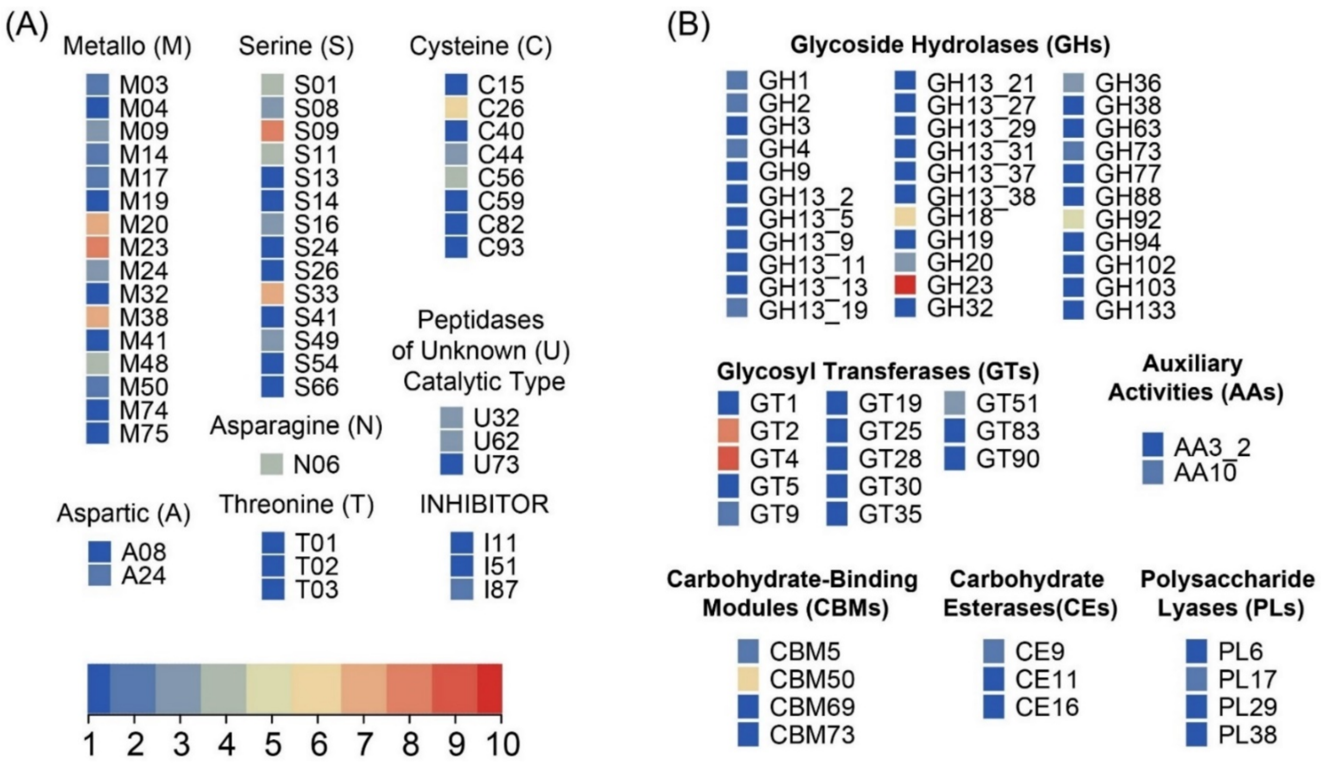

Figure S3. Classification of carbon utilization-related enzymes predicted in WXL538. A, numbers of seven sorts of peptidases and peptidase inhibitors. B, the numbers of each class of Carbohydrate-active enzyme (CAZy). Enzymes were annotated by MEROPS database and dbCAN2 meta server.

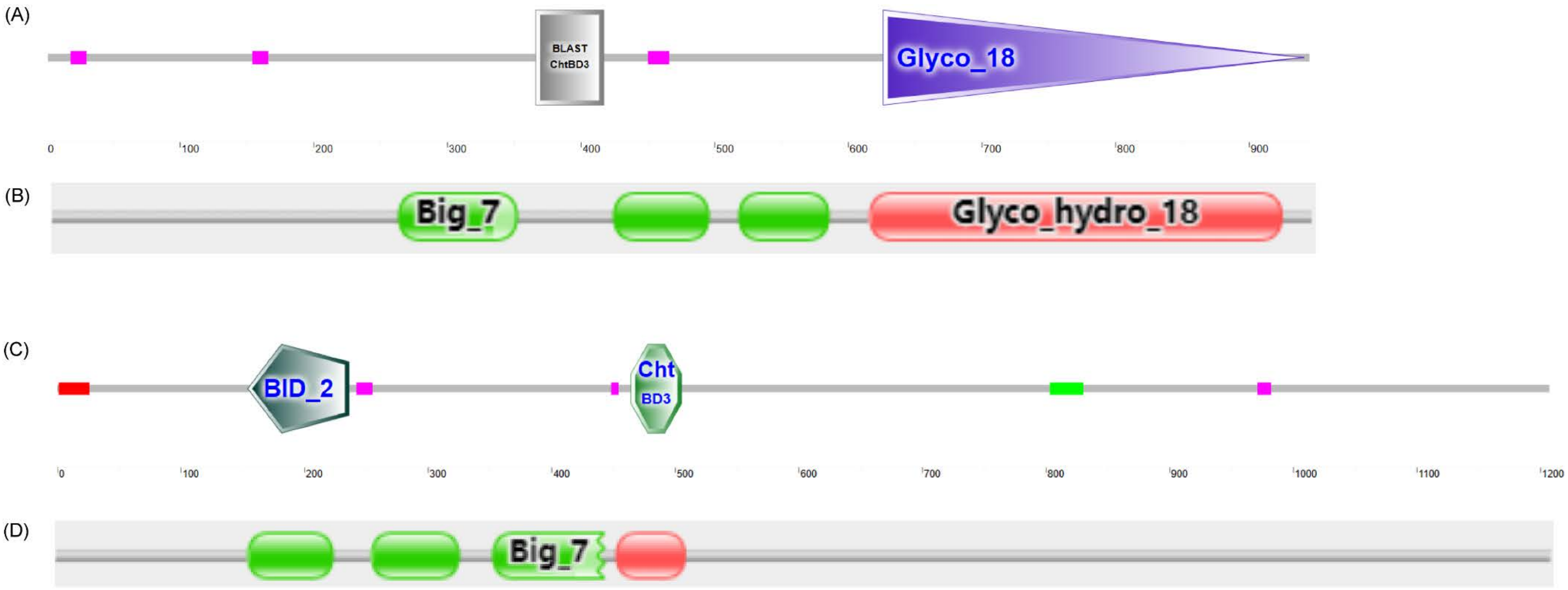

Figure S4. The confident functional domain prediction results of Chi4733 and Chi5174. A, functional domains in Chi4733, predicted with SMART; magenta, low complexity region; ChtBD3, chitin binding domain type 3; Glyco_18, GH18 domain. B, functional domains in Chi4733, predicted with Pfam; green, Big_7, Bacterial Ig domain; Glyco_hydro_18, GH18 domain. C, functional domains in Chi5174, predicted with SMART; red, signal peptide; BID_2, Bacteria Ig-like domain_2; magenta, low complexity region; green, coiled coil region. D, the prediction results of Pfam; green, Big_7; red, CBM_5_12_2, cellulose-binding domain. When predicted with CDD (NCBI), the results were similar to which of Pfam. Only most confident domains were showed in the picture.

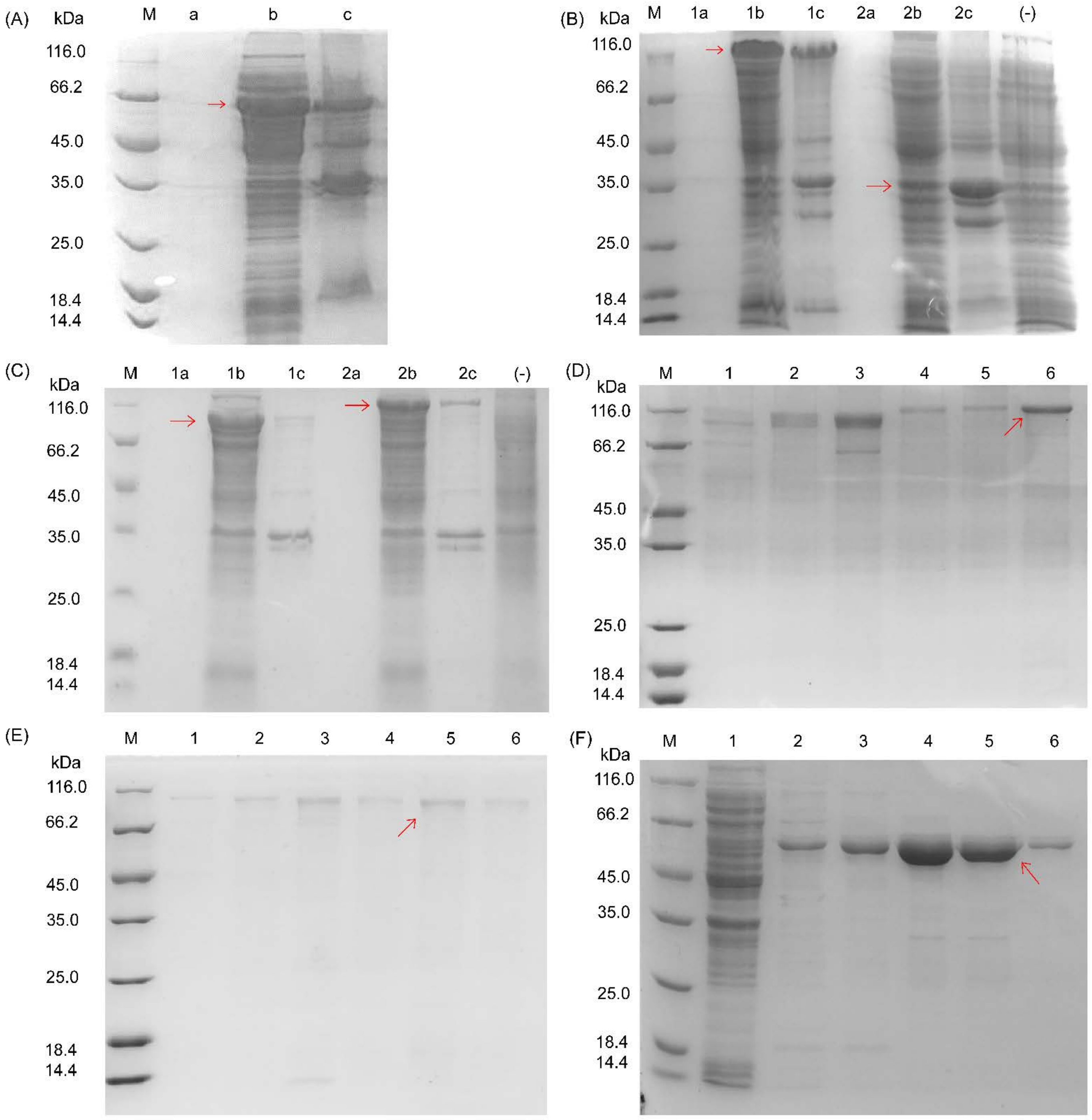

Figure S5. SDS-PAGE of recombinant chitinases. Chi2497 (62.24 kDa), Chi4668 (61.15 kDa), Chi4733 (104.36 kDa), Chi4963 (48.00 kDa), Chi540 (93.06 kDa) and Chi5174 (130.82 kDa) were expressed in *E. coli*. A, Lane 1a, 1b, 1c: the fermentation supernatant, the soluble and the insoluble fraction of induced recombinant plasmid *E. coli* BL21/pET-24a (+)-*chi*2497, respectively. Lane 2a, 2b, 2c: the fermentation supernatant, the soluble and the insoluble fraction of other protein not chitinases, respectively. Lane 3a, 3b: the fermentation supernatant and the soluble fraction of induced recombinant plasmid *E. coli* BL21/pET-24a (+)-*chi*4668, respectively. B, Lane 1a, 1b, 1c: the fermentation supernatant, the soluble and the insoluble fraction of induced recombinant plasmid *E. coli* BL21/pET-24a (+)-*chi*4733, respectively. Lane 2a, 2b, 2c: the fermentation supernatant, the soluble and the insoluble fraction of induced *E. coli* BL21/pET-24a (+)-*chi*4963, respectively. C, Lane 1a, 1b, 1c: the fermentation supernatant, the soluble and the insoluble fraction of induced recombinant plasmid *E.*

coli BL21/pET-24a (+)-*chi*540, respectively. Lane 2a, 2b, 2c: the soluble, the mixture and the insoluble fraction of induced *E. coli* BL21/pET-24a (+)-*chi*5174, respectively. All the recombinants were induced by 0.5 mM IPTG and 15 μl of the mixtures were spotted into the gel. D, Lane 1, 2, 3, 4, 5, 6: recombinant Chi4733 elution washed from Ni-NTA resin at 10, 20, 50, 75, 100, 250 mM imidazole concentration, respectively. E, Lane 1, 2, 3, 4, 5, 6: recombinant Chi540 elution washed from Ni-NTA resin at 10, 20, 50, 75, 100, 250 mM imidazole concentration, respectively. F, Lane 0, the recombinant Chi4668 eluate filtered from Ni-NTA resin. Lane 1, 2, 3, 4, 5, 6: recombinant Chi4668 elution washed from Ni-NTA resin at 10, 20, 50, 75, 100, 250 mM imidazole concentration, respectively. Chi4733 and Chi540 had their best production at 250 mM imidazole concentration, and to balance the purity and yield rate, we kept Chi4668 elution at 100, 250 mM imidazole concentration. M, marker.

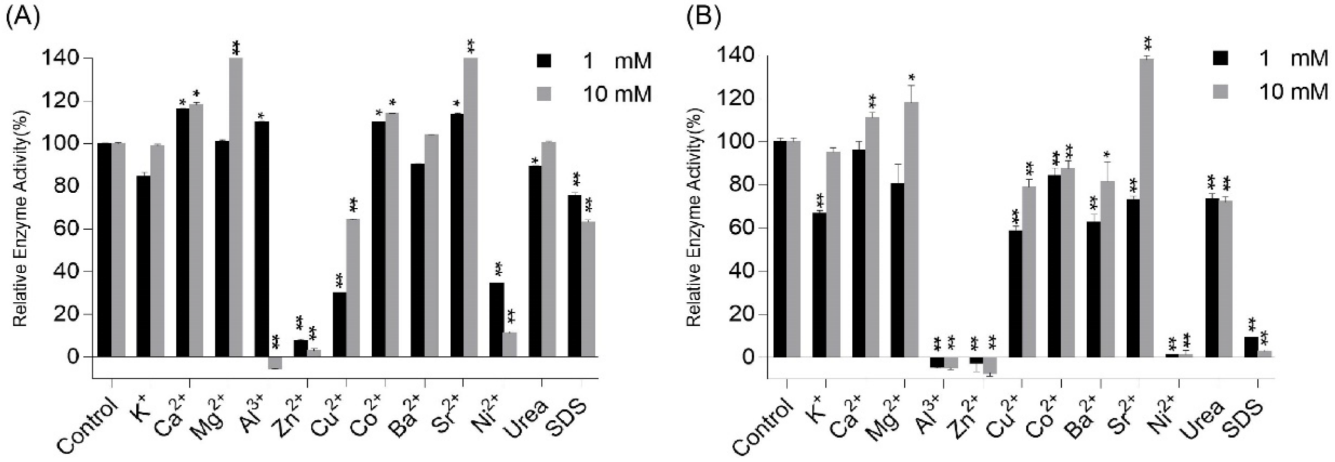

Figure S6. Response of chitinases to metal ions and chemical reagents of different concentration (1 mM and 10 mM). A, results of Chi4733; B, results of Chi540. (** *P* < 0.01; * *P* < 0.05)

Table S1. Chitinases from various *Vibrio* species.

Name	Organism	GenBank accession	CAZY Family	Signal pepe-tide	Molecular weight (kDa)	pI	Optimal temperat-ure(°C)	Optim-al pH	Hydrolysis products	Effect of metal ions
ChiA (Bendt et al., 2001)	*Vibrio* sp. Fi:7	AY007314	GH18	26	79.4	4.16	35	8	N. D.	Ca^{2+} activate,
Chitinase I (Park et al., 2000)	*Vibrio* sp. 98CJ11027	N.M.	N. D.	N. D.	98	N. D.	45	6.0	$(GlcNAc)_2$	Fe^{2+}, Cu^{2+} inhibit
Pa-Chi (Kadokura et al., 2007)	*V. parahaemolyticus* KN1699	N. D.	GH18	N. D.	92	N. D.	50-55	8.0	$(GlcNAc)_2$	N. D.
ChitinaseA (Murao et al., 1992)	*V. alginolyticus* TK-22	N. D.	N. D.	N. D.	66	4.3	45	4，9	$(GlcNAc_{1-5}$	N. D.
Chitinase A (Itoi et al., 2007)	*V. proteolyticus* 442	BAF76068	GH18	26	110	N. D.	N. D.	N. D.	$(GlcNAc)_2$	N. D.
EndoI (Keyhani and Roseman, 1996)	*V. furnissii* NCTC 11218	AAC44673	GH18	N. D.	120	N. D.	35-37	6.5-7.0	$(GlcNAc)_{2-3}$	Mg^{2+} activate, Hg^{2+} inhibit
Chitinase (Zhou et al., 1999)	*Vibrio* sp. 11211	N. D.	GH18	N. D.	30	N. D.	50	6.5	N. D.	N. D.
ChitinaseC1 (Ohishi et al., 1996)	*V. alginolyticus* H-8	N. D.	GH18	N. D.	81	3.9	55	6.5	$(GlcNAc)_{1-3}$	Ag^{+}, Hg^{2+} inhibit

ChitinaseC3(Ohishi et al., 1996)	*V. alginolyticus* H-8	N. D.	GH18	N. D.	68	3.6	50-55	6.5	$(GlcNAc)_{1-6}$	Ag^{+}, Hg^{2+} inhibit
rchi GR52-1 (Zheng et al., 2018)	*Vibrio* sp. GR52	N. D.	GH18	26	87	4.4	50	6	N. D.	Ca^{2+}, Cu^{2+} activate; Mn^{2+}, Co^{2+}, Li^{+}, Hg^{+}, Ag^{+} inhibit
Chi1557 (He et al., 2020)	*V. rotiferianus*	MN5554 66	GH18	23	62	4.3	45-50	5.0-7.0	$(GlcNAc)_{2}$	Ca^{2+} activate; Mn^{2+}, Co^{2+}, Li[illegible] Hg^{+}, Ag^{+} inhibi[illegible]
Chi19 (Honda et al., 2008)	*V. proteolyticus* NBRC 13287	BAE869 96	GH19	22	60	N. D.	40	5.5-7.0	$(GlcNAc)_{2-4}$	N. D.
Vh Nag1 (Suginta et al., 2010)	*V. harveyi* 650	HM1757 15	GH20	N. D.	89	4.9	N. D.	7.5	GlcNAc	N. D.
Vh Nag2 (Suginta et al., 2010)	*V. harveyi* 650	HM1757 16	GH20	N. D.	73	5.4	N. D.	7.0	GlcNAc	N. D.

N.D., no data.

Table S2. Primers used in cloning chitinase and truncated chitinase coding genes. Primers were designed using the Primer-primer 5 design tool. The cleavage sites are underlined.

Primer	Sequence (5'-3')	Restriction site
Chi4733-F	5'-CGCGGATCCATGTATCTAAGAAAGA-3'	EcoR I
Chi4733-R	5'-CCGCTCGAGCTTACTTACATTGATC-3'	Xho I
Chi540-F	5'- CCGGAATTCATGTACAATTGCCAAA-3'	EcoR I
Chi540-R	5'- CCCAAGCTTTTCAGCATTCATTTGA-3'	Hind III
Chi5174-F	5'- CGCGGATCCATGGGAGTGATCACAG-3'	BamH I
Chi5174-R	5'- CCGCTCGAGGCGAGGATCAAGACCT -3'	Xho I
Chi4963-F	5'-CTCGGATCCATGGAGCAAAAAGTCG-3'	BamH I
Chi4963-R	5'-CCGCTCGAGATTCTGCATTTCCTTA-3'	Xho I
Chi4733-232F	5'-AACCGGAATTCATGGGCGCAGCATC-3'	EcoR I
Chi4733-495F	5'-GGCGCGGATCCATGGGTCAATACACAT-3'	BamH I
Chi4733-495R	5'-ACCGCTCGAGGTTTGAACCCAACGT-3'	Xho I
Chi4733-626F	5'- ACCGCTCGAGGTTTGAACCCAACGT -3'	BamH I
Chi4733-942R	5'-CCGCTCGAGGTTTGAACCCAACGTC -3'	Xho I

Table S3. The sensors and regulators responding environment stimulus in WXL538.

Environmental stimulated factor	Sensor/ Regulator	Sequenced ID	Function	Reference
Osmolarity	EnvZ/ OmpR	WXL538_HJONOKIF_03089/ WXL538_HJONOKIF_03090	Porin genes	(Beier and Gross, 2006)
Cationic antimicrobial peptides	CpxA(2)/ CpxR	WXL538_HJONOKIF_00032, WXL538_HJONOKIF_03978/ WXL538_HJONOKIF_00033	Multidrug resistance	(Weatherspoon-Griffin et al., 2014)
copper	CusS/ CusR	WXL538_HJONOKIF_02887/ WXL538_HJONOKIF_02888	Heavy metal tolerance	(Outten et al., 2001)
Phosphate	PhoR/ PhoB	WXL538_HJONOKIF_02686/ WXL538_HJONOKIF_02687	Phosphate absorption	(Lee et al., 1989)
hexose phosphates	UhpB/ UhpA	WXL538_HJONOKIF_04804/ WXL538_HJONOKIF_04803	Glucose-6-phosphate absorption	(Island et al., 1992)
2-ketoglutarate, glutamine	NtrB/ NtrC	WXL538_HJONOKIF_03125/ WXL538_HJONOKIF_03126	Nitrogen utilization	(Jiang and Ninfa, 1999)
Iron	PfeS/ PfeR	WXL538_HJONOKIF_04487/ WXL538_HJONOKIF_04488	Iron acquisition	(Poole and McKay, 2003)
Sensing of oxygen and redox states	ArcB/ ArcA	WXL538_HJONOKIF_02786/ WXL538_HJONOKIF_02784	Quinones reflecting the redox state	(Loui et al., 2009)
Nitrate, nitrite	NarQ/ NarL; NarP	WXL538_HJONOKIF_04410/ WXL538_HJONOKIF_01061; WXL538_HJONOKIF_04411	Nitrate and nitrite respiration	(Noriega et al., 2008)

trimethylamine N-oxide	TorS/ TorR	WXL538_HJONOKIF_04483/ WXL538_HJONOKIF_02198	Anaerobic trimethylamine N-oxide (TMAO) Respiration	(Ansaldi et al., 2001)
C4-dicarboxylate	DctB(2)/ DctD(2)	WXL538_HJONOKIF_03809, WXL538_HJONOKIF_02317/ WXL538_HJONOKIF_04016, WXL538_HJONOKIF_02318	Anaerobic fumarate respiration	(Janausch et al., 2002)
Citrate	CitA/ CitB	WXL538_HJONOKIF_01403/ WXL538_HJONOKIF_01402	Transport and anaerobic metabolism of citrate	(Kaspar et al., 1999;Bott et al., 2010)
tetrathionate	TtrS/ TtrR	WXL538_HJONOKIF_01948/ WXL538_HJONOKIF_01947	Anaerobic tetrathionate respiration	(Price-Carter et al., 2001)
CAI-1	CqsS	WXL538_HJONOKIF_04525	Qorum sensing	(Henke and Bassler, 2004)
AI-1	LuxN	WXL538_HJONOKIF_00979	Qorum sensing	(Henke and Bassler, 2004)
AI-2	LuxP;LuxQ	WXL538_HJONOKIF_04387; WXL538_HJONOKIF_04388	Qorum sensing	(Henke and Bassler, 2004)
Cellular density	VarS/ VarA	WXL538_HJONOKIF_00361/ WXL538_HJONOKIF_01017	Virulence	(Lenz et al., 2005)
Temperature, redox state of quinones, SO_4^{2-}, nicotinic acid	BvgS(2)/ BvgA	WXL538_HJONOKIF_05104, WXL538_HJONOKIF_04579/ WXL538_HJONOKIF_04581	Virulence	(Beier and Gross, 2006)
Chemoattractants	CheA/ CheY(2)	WXL538_HJONOKIF_05169/ WXL538_HJONOKIF_00714, WXL538_HJONOKIF_01875	Chemotaxis	(Miller et al., 2009)

Table S4. Genes related to chemotaxis and flagellum in strain WXL538.

Gene	Ko number	Coding product	Copy number	Sequenced ID
CHEMOTAXIS				
aer	K03776	aerotaxis receptor	1	WXL538_HJONOKIF_05169
cheA	K03407	two-component system, chemotaxis family, sensor kinase CheA [EC:2.7.13.3]	1	WXL538_HJONOKIF_00716
cheB	K03412	two-component system, chemotaxis family, protein-glutamate methylesterase/ glutaminase [EC:3.1.1.61 3.5.1.44]	1	WXL538_HJONOKIF_00717
cheR	K00575	chemotaxis protein methyltransferase CheR [EC:2.1.1.80]	1	WXL538_HJONOKIF_02462
cheV	K03415	two-component system, chemotaxis family, chemotaxis protein CheV	4	WXL538_HJONOKIF_00895, WXL538_HJONOKIF_02463, WXL538_HJONOKIF_05246, WXL538_HJONOKIF_04587
cheW	K03408	purine-binding chemotaxis protein CheW	2	WXL538_HJONOKIF_00719, WXL538_HJONOKIF_00720
cheX	K03409	chemotaxis protein CheX	1	WXL538_HJONOKIF_00168
cheY	K03413	two-component system, chemotaxis family, chemotaxis protein CheY	2	WXL538_HJONOKIF_00714, WXL538_HJONOKIF_01875
cheZ	K03414	chemotaxis protein CheZ	1	WXL538_HJONOKIF_00715
motA	K02556	chemotaxis protein MotA	2	WXL538_HJONOKIF_02567, WXL538_HJONOKIF_04133
motB	K02557	chemotaxis protein MotB	2	WXL538_HJONOKIF_02566, WXL538_HJONOKIF_04132
mcp	K03406	methyl-accepting chemotaxis protein	29	WXL538_HJONOKIF_00062 WXL538_HJONOKIF_00296

WXL538_HJONOKIF_00785
WXL538_HJONOKIF_00959
WXL538_HJONOKIF_01064
WXL538_HJONOKIF_01076
WXL538_HJONOKIF_01515
WXL538_HJONOKIF_01609
WXL538_HJONOKIF_01714
WXL538_HJONOKIF_01963
WXL538_HJONOKIF_02053
WXL538_HJONOKIF_02087
WXL538_HJONOKIF_02225
WXL538_HJONOKIF_02262
WXL538_HJONOKIF_02847
WXL538_HJONOKIF_02925
WXL538_HJONOKIF_03057
WXL538_HJONOKIF_03758
WXL538_HJONOKIF_03899
WXL538_HJONOKIF_04418
WXL538_HJONOKIF_04451
WXL538_HJONOKIF_04585
WXL538_HJONOKIF_04709
WXL538_HJONOKIF_04995
WXL538_HJONOKIF_05202
WXL538_HJONOKIF_05257
WXL538_HJONOKIF_05392
WXL538_HJONOKIF_05404

				WXL538_HJONOKIF_05437
FLAGELLUM				
flgA	K02386	flagellar basal body P-ring formation protein FlgA	2	WXL538_HJONOKIF_02464, WXL538_HJONOKIF_04092
flgB	K02387	flagellar basal-body rod protein FlgB	2	WXL538_HJONOKIF_02461, WXL538_HJONOKIF_04093
flgC	K02388	flagellar basal-body rod protein FlgB	2	WXL538_HJONOKIF_02460, WXL538_HJONOKIF_04094
flgD	K02389	flagellar basal-body rod modification protein FlgD	2	WXL538_HJONOKIF_02459, WXL538_HJONOKIF_04095
flgE	K02390	flagellar hook protein FlgE	2	WXL538_HJONOKIF_02458, WXL538_HJONOKIF_04096
flgF	K02391	flagellar basal-body rod protein FlgF	2	WXL538_HJONOKIF_02457, WXL538_HJONOKIF_04097
flgG	K02392	flagellar basal-body rod protein FlgG	2	WXL538_HJONOKIF_02456, WXL538_HJONOKIF_04098
flgH	K02393	flagellar L-ring protein FlgH	2	WXL538_HJONOKIF_02455, WXL538_HJONOKIF_04099
flgI	K02394	flagellar P-ring protein FlgI	2	WXL538_HJONOKIF_02454, WXL538_HJONOKIF_04100
flgJ	K02395	peptidoglycan hydrolase FlgJ	2	WXL538_HJONOKIF_02453, WXL538_HJONOKIF_04101
flgK	K02396	flagellar hook-associated protein 1	2	WXL538_HJONOKIF_02452, WXL538_HJONOKIF_04102
flgL	K02397	flagellar hook-associated protein 3 FlgL	2	WXL538_HJONOKIF_02451, WXL538_HJONOKIF_04103

flgM	K02398	negative regulator of flagellin synthesis FlgM	2	WXL538_HJONOKIF_02465, WXL538_HJONOKIF_04091
flgN	K02399	flagellar biosynthesis protein FlgN	2	WXL538_HJONOKIF_02466, WXL538_HJONOKIF_04090
flgP	K09860	outer membrane protein FlgP	1	WXL538_HJONOKIF_02467
flgO	K24344	flagellar H-ring protein FlgO	1	WXL538_HJONOKIF_02468
flgT	K24343	flagellar H-ring protein FlgT	1	WXL538_HJONOKIF_02469
flhA	K02400	flagellar biosynthesis protein FlhA	2	WXL538_HJONOKIF_00710, WXL538_HJONOKIF_04143
flhB	K02401	flagellar biosynthesis protein FlhB	2	WXL538_HJONOKIF_00709, WXL538_HJONOKIF_04144
fliA	K02405	RNA polymerase sigma factor FliA	2	WXL538_HJONOKIF_00710, WXL538_HJONOKIF_04143
fliC	K02406	flagellar assembly protein, flagellin	7	WXL538_HJONOKIF_00684, WXL538_HJONOKIF_00685, WXL538_HJONOKIF_00686, WXL538_HJONOKIF_02448, WXL538_HJONOKIF_02449, WXL538_HJONOKIF_02450, WXL538_HJONOKIF_04141
fliD	K02407	flagellar hook-associated protein 2	2	WXL538_HJONOKIF_00688, WXL538_HJONOKIF_04139
fliE	K02408	flagellar hook-basal body complex protein FliE	2	WXL538_HJONOKIF_00694, WXL538_HJONOKIF_04152
fliF	K02409	flagellar M-ring protein FliF	2	WXL538_HJONOKIF_00695, WXL538_HJONOKIF_04153

fliG	K02410	flagellar motor switch protein FliG	2	WXL538_HJONOKIF_00696, WXL538_HJONOKIF_04154
fliH	K02411	flagellar assembly protein FliH	2	WXL538_HJONOKIF_00697, WXL538_HJONOKIF_04155
fliI	K02412	flagellum-specific ATP synthase [EC:7.4.2.8]	2	WXL538_HJONOKIF_00698, WXL538_HJONOKIF_04156
fliJ	K02413	flagellar protein FliJ	1	WXL538_HJONOKIF_00699
fliK	K02414	flagellar hook-length control protein FliK	2	WXL538_HJONOKIF_00701, WXL538_HJONOKIF_04136
fliL	K02415	flagellar protein FliL	3	WXL538_HJONOKIF_00702, WXL538_HJONOKIF_03369, WXL538_HJONOKIF_04135
fliM	K02416	flagellar motor switch protein FliM	1	WXL538_HJONOKIF_00703
fliN	K02417	flagellar motor switch protein FliN	2	WXL538_HJONOKIF_00704, WXL538_HJONOKIF_04148
fliO, *fliZ*	K02418	flagellar protein FliO/FliZ	2	WXL538_HJONOKIF_00705
fliP	K02419	flagellar biosynthesis protein FliP	1	WXL538_HJONOKIF_00706, WXL538_HJONOKIF_04147
fliQ	K02420	flagellar biosynthesis protein FliQ	2	WXL538_HJONOKIF_00707, WXL538_HJONOKIF_04146
fliR	K02421	flagellar biosynthesis protein FliR	2	WXL538_HJONOKIF_00708, WXL538_HJONOKIF_04145
fliS	K02422	flagellar secretion chaperone FliS	2	WXL538_HJONOKIF_00690, WXL538_HJONOKIF_04138
flrA	K10941	sigma-54 dependent transcriptional regulator, flagellar regulatory protein	1	WXL538_HJONOKIF_00691

flrC	K10943	two-component system, response regulator FlrC	2	WXL538_HJONOKIF_00693, WXL538_HJONOKIF_04151
motX	K21217	sodium-type polar flagellar protein MotX	1	WXL538_HJONOKIF_00090
motY	K21218	sodium-type flagellar protein MotY	2	WXL538_HJONOKIF_00819, WXL538_HJONOKIF_04150

Table S5. The confidently predicted domains or features of putative chitinase in *V. harveyi* WXL538.

**Chitinase**	**Predict conserved domain**
Chi4963	Signal peptide (1-23) + GH18 (23-416)
Chi4733	Ig-like domain (269-338, 432-501, 527-593) + GH18 (626-945)
Chi540	Signal peptide (1-22) + ChtBD3 (28-74) + Ig-like domain (182-247, 339-405, 430-496) + GH18 (524-825)
Chi4668	Signal peptide (1-23) + GH18 (32-317) + Ig-like domain (352-424, 429-501) + ChtBD3 (507-557)
Chi3480	Signal peptide (1-21) + ChiA (N-terminal) (21-155) + GH18 (159-575) + PKD (600-690, 701-787) + ChtBD3 (795-840)
Chi44930	Signal peptide (1-30) + ChtBD3 (37-82) + Ig-like domain (209-261) + GH18 (322-782) + ChiC (C-terminal) (810-979) + ChtBD3 (1009-1051)
Chi2497	Signal peptide (1-21) + GH19 (100-338) + ChtBD3 (436-478, 516-561)
Chi5174	Signal peptide (1-26) + Ig-like domain (160-224, 259-326, 356-420) + ChtBD3 (464-505) + CBM5_12_2 (927-975, 990-1004)

Ig-like: immunoglobulin-like; CBM: carbohydrate-binding module; ChtBD: chitin-binding domain; PKD: Polycystic Kidney Disease

Reference

Ansaldi, M., Jourlin-Castelli, C., Lepelletier, M., Theraulaz, L., and Mejean, V. (2001). Rapid dephosphorylation of the TorR response regulator by the TorS unorthodox sensor in *Escherichia coli*. *J Bacteriol* 183**,** 2691-2695. doi: 10.1128/JB.183.8.2691-2695.2001.

Beier, D., and Gross, R.J.C.O.I.M. (2006). Regulation of bacterial virulence by two-component systems. *Current Opinion in Microbiology* 9**,** 143-152. doi: 10.1016/j.mib.2006.01.005.

Bendt, A., Hüller, H., Kammel, U., Helmke, E., and Schweder, T.J.E. (2001). Cloning, expression, and characterization of a chitinase gene from the Antarctic psychrotolerant bacterium *Vibrio* sp. strain Fi:7. *Extremophiles* 5**,** 119-126. doi: 10.1007/s007920100179.

Bott, M., Meyer, M., and Dimroth, P.J.M.M. (2010). Regulation of anaerobic citrate metabolism in *Klebsiella pneumoniae*. *Molecular Microbiology* 18**,** 533-546. doi: 10.1111/j.1365-2958.1995.mmi_18030533.x.

He, X., Yu, M., Wu, Y., Ran, L., Liu, W., and Zhang, X.H. (2020). Two highly similar chitinases from marine *Vibrio* species have different enzymatic properties. *Mar Drugs* 18. doi: 10.3390/md18030139.

Henke, J.M., and Bassler, B.L. (2004). Three parallel quorum-sensing systems regulate gene expression in *Vibrio harveyi*. *J Bacteriol* 186**,** 6902-6914. doi: 10.1128/JB.186.20.6902-6914.2004.

Honda, Y., Taniguchi, H., and Kitaoka M.J. (2008). A reducing-end-acting chitinase from *Vibrio proteolyticus* belonging to glycoside hydrolase family 19. *Appl Microbiol Biotechnol* 78**,** 627. doi: 10.1007/s00253-008-1352-2.

Island, M.D., Wei, B., and Kadner, R.J.J. (1992). Structure and function of the uhp genes for the sugar phosphate transport system in *Escherichia coli* and *Salmonella typhimurium*. *Journal of Bacteriology* 174**,** 2754-2762. doi: 10.1128/jb.174.9.2754-2762.1992.

Itoi, S., Kanomata, Y., Koyama, Y., Kadokura, K., Uchida, S., Nishio, T., Oku, T., and

Sugita, H. (2007). Identification of a novel endochitinase from a marine bacterium *Vibrio proteolyticus* strain No. 442. *Biochim Biophys Acta* 1774**,** 1099-1107. doi: 10.1016/j.bbapap.2007.06.003.

Janausch, I., Zientz, E., Tran, Q., Kröger, A., and Unden, G.J. (2002). C4-dicarboxylate carriers and sensors in bacteria. *Biochimica et Biophysica Acta (BBA)-Bioenergetics* 1553**,** 39-56. doi: 10.1016/S0005-2728(01)00233-X.

Jiang, P., and Ninfa, A.J. (1999). Regulation of Autophosphorylation of *Escherichia coli* Nitrogen Regulator II by the PII Signal Transduction Protein. *Journal of Bacteriology* 181**,** 1906-1911. doi: 10.1128/JB.181.6.1906-1911.1999.

Kadokura, K., Rokutani, A., Yamamoto, M., Ikegami, T., Sugita, H., Itoi, S., Hakamata, W., Oku, T., and Nishio, T. (2007). Purification and characterization of *Vibrio parahaemolyticus* extracellular chitinase and chitin oligosaccharide deacetylase involved in the production of heterodisaccharide from chitin. *Appl Microbiol Biotechnol* 75**,** 357-365. doi: 10.1007/s00253-006-0831-6.

Kaspar, S., Perozzo, R., Reinelt, S., Meyer, M., Pfister, K., Scapozza, L., and Bott, M.J. (1999). The periplasmic domain of the histidine autokinase CitA functions as a highly specific citrate receptor. *Molecular Microbiology* 33**,** 858-872. doi: 10.1046/j.1365-2958.1999.01536.x.

Keyhani, N.O., and Roseman, S.J. (1996). The chitin catabolic cascade in the marine bacterium *Vibrio furnissii*: molecular cloning, isolation, and characterization of a periplasmic chitodextrinase. 271**,** 33414-33424. doi: 10.1074/jbc.271.52.33414.

Lee, T.-Y., Makino, K., Shinagawa, H., Amemura, M., and Nakata, A.J. (1989). Phosphate regulon in members of the family *Enterobacteriaceae*: comparison of the phoB-phoR operons of *Escherichia coli*, *Shigella dysenteriae*, and *Klebsiella pneumoniae*. *Journal of Bacteriology* 171**,** 6593-6599. doi: 10.1128/jb.171.12.6593-6599.1989.

Lenz, D.H., Miller, M.B., Zhu, J., Kulkarni, R.V., and Bassler, B.L. (2005). CsrA and three redundant small RNAs regulate quorum sensing in *Vibrio cholerae*. *Mol Microbiol* 58**,** 1186-1202. doi: 10.1111/j.1365-2958.2005.04902.x.

Loui, C., Chang, A.C., and Lu, S. (2009). Role of the ArcAB two-component system in the resistance of *Escherichia coli* to reactive oxygen stress. *BMC Microbiol* 9**,** 183. doi: 10.1186/1471-2180-9-183.

Miller, L.D., Russell, M.H., and Alexandre, G. (2009). "Diversity in Bacterial Chemotactic Responses and Niche Adaptation," in *Advances in Applied Microbiology, Vol 66,* eds. A.L. Laskin, S. Sariaslani & G. Gadd. (San Diego: CA: Elsevier Academic Press Inc), 53-75.

Murao, S., Kawada, T., Itoh, H., Oyama, H., and Shin, T. (1992). Purification and Characterization of a Novel Type of Chitinase from *Vibrio alginolyticus*TK-22. *Bioscience, Biotechnology, and Biochemistry* 56**,** 368-369. doi: 10.1271/bbb.56.368.

Noriega, C.E., Schmidt, R., Gray, M.J., Chen, L.L., and Stewart, V. (2008). Autophosphorylation and dephosphorylation by soluble forms of the nitrate-responsive sensors NarX and NarQ from *Escherichia coli* K-12. *J Bacteriol* 190**,** 3869-3876. doi: 10.1128/JB.00092-08.

Ohishi, K., Yamagishi, M., Ohta, T., Suzuki, M., Izumida, H., Sano, H., Nishijima, M., Tan, M.J.J.O.F., and Bioengineering (1996). Purification and properties of two chitinases from *Vibrio alginolyticus* H-8. 82**,** 598-600. doi: 10.1016/S0922-338X(97)81260-3.

Outten, F.W., Huffman, D.L., Hale, J.A., and O'halloran, T.V. (2001). The independent cue and cus systems confer copper tolerance during aerobic and anaerobic growth in *Escherichia coli. J Biol Chem* 276**,** 30670-30677. doi: 10.1074/jbc.M104122200.

Park, S.H., Lee, J.H., and Lee, H.K.J. (2000). Purification and characterization of chitinase from a marine bacterium, *Vibrio* sp. 98CJ11027. *Journal of Microbiology* 38**,** 224-229. doi: Not available.

Poole, K., and Mckay, G.a.J.F.B. (2003). Iron acquisition and its control in *Pseudomonas aeruginosa*: many roads lead to Rome. *Frontiers in Bioscience A Journal & Virtual Library* 8**,** 661-686. doi: 10.2741/1051.

Price-Carter, M., Tingey, J., Bobik, T.A., and Roth, J.R. (2001). The alternative electron acceptor tetrathionate supports B12-dependent anaerobic growth of

Salmonella enterica serovar typhimurium on ethanolamine or 1,2-propanediol. *J Bacteriol* 183**,** 2463-2475. doi: 10.1128/JB.183.8.2463-2475.2001.

Suginta, W., Chuenark, D., Mizuhara, M., and Fukamizo, T. (2010). Novel beta-N-acetylglucosaminidases from *Vibrio harveyi* 650: cloning, expression, enzymatic properties, and subsite identification. *BMC Biochem* 11**,** 40. doi: 10.1186/1471-2091-11-40.

Weatherspoon-Griffin, N., Yang, D., Kong, W., Hua, Z., and Shi, Y.J.J.O.B.C. (2014). The CpxR/CpxA two-component regulatory system up-regulates the multidrug resistance cascade to facilitate *Escherichia coli* resistance to a model antimicrobial peptide. *Journal of Biological Chemistry* 289**,** 32571-32582. doi: 10.1074/jbc.M114.565762.

Zheng, J.M., Liang, Y.H., Zhu, F., Xiu-Yun, Y.E., and Lin, J.J. (2018). Cloning,expression and characterization of the chitinase gene from *Vibrio* sp. GR52. *Microbiology Communication*. doi: 10.13344/j.microbiol.china.170571.

Zhou, S.N., Yang, C.Y., Lu, Y.J., Huang, L., Cai, C.H. and Lin, Y.C. (1999). Isolation and characterization of chitinase from a marine bacterium *Vibrio* sp. *World Journal of Microbiology and Biotechnology* 15**,** 745-746. doi: 10.1023/A:1008932217926.
